# Combination of percutaneous thermal ablation and adoptive Th9 cell transfer therapy against non-small cell lung cancer

**DOI:** 10.1186/s40164-024-00520-8

**Published:** 2024-05-17

**Authors:** Hanbo Pan, Yu Tian, Siyu Pei, Wanlin Yang, Yanyang Zhang, Zenan Gu, Hongda Zhu, Ningyuan Zou, Jiaqi Zhang, Long Jiang, Yingjie Hu, Shengping Shen, Kai Wang, Haizhen Jin, Ziming Li, Yanyun Zhang, Yichuan Xiao, Qingquan Luo, Hui Wang, Jia Huang

**Affiliations:** 1 Department of Thoracic Surgical Oncology, Shanghai Lung Cancer Center, Shanghai Chest Hospital, Shanghai Jiao Tong University School of Medicine, Shanghai, 200030 China; 2 Chinese Academy of Sciences Key Laboratory of Tissue Microenvironment and Tumor, Shanghai Institute of Nutrition and Health, University of Chinese Academy of Sciences, Chinese Academy of Sciences, Shanghai, 200030 China; 3 Department of Oncology, Shanghai Lung Cancer Center, Shanghai Chest Hospital, Shanghai Jiao Tong University School of Medicine, Shanghai, 200030 China; 4 Department of Central Laboratory, Shanghai Chest Hospital, Shanghai Jiao Tong University School of Medicine, Shanghai, 200030 China

**Keywords:** Non-small cell lung cancer, Percutaneous thermal ablation, Adoptive Th9 cell therapy, Tumor immunotherapy, Patient-derived xenograft

## Abstract

**Background:**

Non-small cell lung cancer (NSCLC) is one of the predominant malignancies globally. Percutaneous thermal ablation (PTA) has gained widespread use among NSCLC patients, with the potential to elicit immune responses but limited therapeutic efficacies for advanced-stage disease. T-helper type 9 (Th9) cells are a subset of CD4^+^ effector T cells with robust and persistent anti-tumor effects. This study proposes to develop PTA-Th9 cell integrated therapy as a potential strategy for NSCLC treatment.

**Methods:**

The therapeutic efficacies were measured in mice models with subcutaneously transplanted, recurrence, or lung metastatic tumors. The tumor microenvironments (TMEs) were evaluated by flow cytometry. The cytokine levels were assessed by ELISA. The signaling molecules were determined by quantitative PCR and Western blotting. The translational potential was tested in the humanized NSCLC patient-derived xenograft (PDX) model.

**Results:**

We find that PTA combined with adoptive Th9 cell transfer therapy substantially suppresses tumor growth, recurrence, and lung metastasis, ultimately extending the survival of mice with NSCLC grafts, outperforming both PTA and Th9 cell transfer monotherapy. Analysis of TMEs indicates that combinatorial therapy significantly augments tumor-infiltrating Th9 cells, boosts anti-tumor effects of CD8^+^ T cells, and remodels tumor immunosuppressive microenvironments. Moreover, combinatorial therapy significantly strengthens the regional and circulation immune response of CD8^+^ T cells in mice with tumor lung metastasis and induces peripheral CD8^+^ T effector memory cells in mice with tumor recurrence. Mechanically, PTA reinforces the anti-tumor ability of Th9 cells primarily through upregulating interleukin (IL)-1β and subsequently activating the downstream STAT1/IRF1 pathway, which could be effectively blocked by intercepting IL-1β signaling. Finally, the enhanced therapeutic effect of combinatorial therapy is validated in humanized NSCLC PDX models.

**Conclusions:**

Collectively, this study demonstrates that combinatorial therapy displays robust and durable anti-tumor efficacy and excellent translational potential, offering excellent prospects for translation and emerging as a promising approach for NSCLC treatment.

**Supplementary Information:**

The online version contains supplementary material available at 10.1186/s40164-024-00520-8.

## Background

With an estimated 2.2 million newly diagnosed cases and over 1.7 million deaths yearly, lung cancer (LC) remains the most prevalent malignancy and the leading cause of cancer-related mortalities globally, and non-small-cell lung cancer (NSCLC) accounts for about 80-85% of total LC morbidity [1]. Despite the implementation of screening programs, at least 30% of NSCLC cases are diagnosed at an advanced stage, rendering surgical resection no longer the preferred treatment approach [2]. Regrettably, conventional methods such as platinum-based chemotherapy and radiotherapy exhibit limited efficacy, leading to a poor prognosis for patients with advanced NSCLC [3, 4]. Adoptive cell therapy (ACT) has emerged as one of the most potent cancer immunotherapy strategies with excellent specificity [5]. Nonetheless, the dense, complex extracellular matrix of tumors, along with low chemokine expression levels and the scarcity of immunogenic tumor neoantigens, significantly impede the recognition and infiltration of transferred T cells into tumors [5, 6]. Additionally, the tumor microenvironment (TME) harbors a multitude of immunosuppressive elements, including a variety of immunosuppressive cells (e.g., regulatory T cells (Tregs), tumor-associated macrophages (TAMs), and myeloid-derived suppressor cells (MDSCs)), inhibitory receptors on tumor cells (e.g., programmed death-ligand 1 (PD-L1) and cytotoxic T lymphocyte-associated antigen 4 (CTLA-4)), and hypoxic conditions. These factors collectively diminish the anti-tumor effectiveness of transferred T cells, leading to their exhaustion and resulting in the limited efficacy of ACT for treating solid malignancies [7]. Moreover, in the case of ‘cold’ tumors like NSCLC characterized by inadequate immune cell infiltration, transferred T cells face challenges infiltrating the TME, leading to suboptimal ACT outcomes [8]. Given this, an urgent imperative exists to introduce immune-stimulation strategies to enhance the therapeutic efficacy of ACT for advanced NSCLC [5]. Given this, an urgent imperative exists to introduce immune-stimulation strategies to enhance the therapeutic efficacy of ACT for advanced NSCLC.

Percutaneous thermal ablation (PTA) encompasses hyperthermic techniques and cryoablation, with the former primarily comprising radiofrequency ablation (RFA) and microwave ablation (MWA). PTA has been widely utilized for early-stage NSCLC, exhibiting superiorities over surgical resection in preserving more normal lung tissue and reducing comorbidities [9]. Additionally, PTA can address primary and metastatic lesions in advanced NSCLC patients for whom conventional therapy is unsuitable, thereby extending their survival [10]. By generating an extremely high temperature within the tumor’s targeted area, hyper-thermic PTA achieves irreversible cellular damage and, ultimately, cell death and tissue coagulation necrosis [11]. During this process, tumor-specific antigens (TSAs) are liberated from tumor cells, thereby recruiting immune cells, such as lymphocytes, dendritic cells (DCs), and macrophages (Mφ) [12]. However, in advanced NSCLC patients, PTA often fails to eradicate tumor lesions completely, resulting in tumor recurrence or progression shortly following treatment [13]. Considering the potential of PTA to stimulate anti-tumor immune responses, a novel PTA-integrated therapy, combining PTA with ACT to overcome the transient limitations of PTA in NSCLC, represents a promising strategy for achieving a potent anti-tumor approach.

In recent years, T-helper type 9 (Th9) cells, a unique subset of CD4^+^ effector T cells, have emerged as a promising avenue against advanced malignancies, attributed mainly to their robust and enduring anti-tumor effects, primarily driven by interleukin (IL)-9 secretion [14–16]. Compared to conventional Th subsets, Th9 cells exhibit lower exhaustion levels while demonstrating cytolytic activity similar to Th1 cells and persisting similarly to ‘stem cell-like’ Th17 cells in vivo, thus generating a strong anti-tumor effect [17]. Th9 cells also demonstrate heightened efficacy against ‘cold tumors’ compared to ordinary CD8^+^ T cells [18]. Moreover, in an acute lymphocytic leukemia model, chimeric antigen receptor (CAR) T9 cells display markedly greater and enduring anti-tumor efficacy when compared to CAR T1 cells [19]. Additionally, adoptive Th9 cell transfer therapy effectively inhibits lung metastasis of melanoma and prolongs the survival of mice via recruiting DCs [14]. These findings indicate that Th9 cell-mediated ACT holds promise as a therapeutic approach for solid tumors. However, the utilization of Th9 cells combined with clinically relevant immune-activation modalities, which could potentially develop a novel therapeutic strategy with enhanced efficacy, has rarely been documented.

Following PTA, pro-inflammatory cytokines are released from the ablated tissues, tumor cells, and immune cells, leading to increased levels of IL-1β, tumor necrosis factor (TNF)-α, IL-6, and IL-8 on a timescale of hours to days [12, 20]. Intriguingly, pro-inflammatory cytokines, especially IL-1β, TNF-α, and IL-33, have been found to dramatically boost the proliferation, cytokine production, and tumor-eliminating potency of Th9 cells [21–23]. Given this, we postulated that PTA could promote the effects of Th9 cells in eliminating NSCLC tumors, and thus, in this study, we explore the therapeutic potential of combining PTA with adoptive Th9 cell transfer. Our findings reveal that this combinatorial approach significantly suppresses NSCLC tumor growth, recurrence, lung metastasis, and extends the survival of mice bearing NSCLC grafts, offering a compelling therapeutic strategy with substantial translational prospects in NSCLC treatment.

## Methods

### Animals

C57BL/6 (male, 4–5 weeks old) and immunodeficient nonobese diabetic/ShiLtJGpt-*Prkdc*^em26Cd52^*Il2rg*^em26Cd22^/Gpt (NTG) mice (male, 4–5 weeks old) were purchased from SPF (Beijing) Biotechnology Co., Ltd. (Beijing, China) and stabilized for 2 weeks before experiments. IL-9-internal ribosome entry site (IRES)-EGFP mice were created by Biocytogen Co., Ltd. (Beijing, China), utilizing the CRISPR/Cas9 system to insert an IRES-EGFP-SV-pA sequence before the termination codon of the *Il9* gene through CRISPR/Cas9, as reported by our team previously [24]. This genetic engineering technique allows for the expression of EGFP as a reporter under the control of the *Il9* gene’s regulatory elements, facilitating the study of IL-9 expression in various biological contexts. All mice were bred and housed under specific-pathogen-free (SPF) conditions at the Animal Center of Shanghai Chest Hospital. For animal experiments, mice were randomly divided into different groups and treated specifically. All animal experiments were carried out following the National Institutes of Health Guide for the Care and Use of Laboratory Animals and were approved by the institutional biomedical research ethics committee of Shanghai Jiao Tong University. All animal studies have followed the ARRIVE guidelines.

### Antibodies and reagents

For the flow cytometric analysis, anti-mouse CD45 (I3/2.3), anti-mouse CD3 (17A2), anti-mouse CD11c (N418), anti-mouse NK1.1 (S17016D), anti-mouse CD45R/B220 (M1/70), anti-mouse CD4 (RM4-5), anti-mouse CD8 (53 − 6.7), anti-mouse CD62L (MEL-14), anti-mouse interferon (IFN)-γ (XMG1.2), anti-mouse IL-4 (11B11), anti-mouse IL-9 (RM9A4), anti-mouse CD25 (MF-14), anti-mouse Granzyme B (QA16A02), anti-mouse PD-1 (RMP1-30), anti-mouse F4/80 (QA17A29), anti-mouse MHC-II (M5/114.15.2), anti-mouse CD206 (C068C2), anti-mouse Ly-6G (1A8), anti-mouse CD127 (S18006K), anti-mouse IL-10 (JES5-16E3), anti-mouse GITR (DTA-1), anti-mouse CTLA-4 (UC10-4B9), anti-human CD45 (HI30), anti-human CD3 (OKT3), anti-human CD4 (A161A1), anti-human HLA-DR (L243), anti-human IL-9 (MH9A4), anti-human PD-1 (EH12.2H7), anti-human Granzyme B (QA16A02), and Zombie UV Fixable Viability Kit were purchased from BioLegend. Anti-mouse CD44 (IM7), anti-mouse Ly6C (HK1.4), and anti-mouse FOXP3 (FJK-16s) were purchased from eBioscience. Anti-mouse CD11b (M1/70) was purchased from BD Pharmingen. For the Western blot assay, signal transducer and activator of transcription 1 (STAT1, D1K9Y), p-STAT1 (Tyr701; 58D6), interferon regulatory factor (IRF) 1 (D5E4), β-actin (D6A8), and HRP-linked anti-rabbit IgG were purchased from Cell Signaling Technology (Danvers, MA, USA).

Cisplatin, Raleukin (also known as anakinra), SU6656, and cyanidin 3-O-glucoside chloride (C3G) were purchased from MedChemExpress (Monmouth Junction, NJ, USA). Purified anti-mouse IL-1β monoclonal antibody (mAb), anti-mouse TNFR type 1 mAb, anti-mouse TNFR type 2 mAb, and IgG isotype control were purchased from Biolegend. Recombinant mouse transforming growth factor (TGF)-β1 was purchased from Biolegend, recombinant mouse IL-4, human IL-4, and human TGF-β were purchased from Novoprotein (Jiangsu, China), and InVivoPlus anti-mouse IFN-γ antibodies were purchased from Leinco Technologies (St. Louis, Missouri, USA). Dulbecco’s modified Eagle’s medium (DMEM; with high glucose, 4.0 mM L-glutamine, and sodium pyruvate; SH30243.01) and Roswell Park Memorial Institute (RPMI) 1640 medium modified (SH30809.01) were purchased from Hyclone (Logan, UT, United States). Fetal bovine serum (FBS; 10,270) and penicillin/streptomycin (15140-122) were purchased from Gibco. Levofloxacin was from Absin Bioscience Inc., and Ficoll and Percoll were from MP Biomedicals (Santa Ana, California, USA). DNase I and Collagenase II and IV were purchased from STEMCELL Technologies. Mouse and human CD4 and CD45 microbeads were purchased from Miltenyi Biotec (Bergisch Gladbach, Cologne, Germany).

### Cell line culture

Mice Lewis lung cancer (LLC)/Luciferase (LUC) cell line was a generous gift from Dr. Feng Yao (Shanghai Chest Hospital). Mice LLC and LLC/LUC and human embryonic kidney HEK293T cell lines were cultured in DMEM supplemented with 10% FBS, 100 U/mL penicillin, and 100 mg/mL streptomycin. The cell lines were cultivated at 37 °C with 5% CO_2_ and passaged when the cell intensity was approximately 70–80%. The mycoplasma contamination of all cells used in the study was conventionally tested using Mycoplasma Detection Kits and proved negative.

### In vitro Th9 cell culture and differentiation

The in vitro Th9 cell polarization was conducted according to previously described protocol [24]. For in vitro mice Th9 cell differentiation, the primary lymphocytes were separated from the lymph nodes, and spleens of mice, and CD4^+^ T cells were further enriched through negative selection by using the MojoSort Mouse CD4^+^ T Cell Isolation Kit (BioLegend) per the instructions from the manufacturer, followed by purification of naive CD4^+^ T cells (CD4^+^CD44^lo^CD62L^hi^ cells) by using flow cytometry. Naive CD4^+^ T cells were stimulated in the round bottom 96-well cell culture plate (Corning Incorporated, Kennebunk, ME, USA) with plate-bound anti-mouse CD3ε (5 µg/mL) and anti-mouse CD28 (5 µg/mL) antibodies and T cell culture medium (RPMI 1640, 10% FBS, 1 × nonessential amino acids, 100 U/mL penicillin, 100 mg/mL streptomycin, 1 × levofloxacin, and 50 µM β-mercaptoethanol). Naïve CD4^+^ T cells were differentiated into Th9 cells with anti-mouse IFN-γ (10 µg/mL), anti-mouse IL-4 (20 ng/mL), and anti-mouse TGF-β1 (10 ng/mL) and were used as effector CD4^+^ T cells (Th0 cells) via cultured without supplemented with TGF-β1 and IL-4. Th9 or Th0 cells were routinely harvested after differentiation for 3–4 days, and the cytokine expression was detected by flow cytometry or qPCR.

The total tumor proteins and cytokines levels in the supernatant of tumor digests were measured by Pierce BCA Protein Assay Kit and ELISA, respectively. To explore the pro-inflammatory cytokines induced by PTA that contributed to the differentiation of Th9 cells, naïve CD4^+^ T cells were polarized in the round-bottom 96-well palates under the Th9-differentiation condition with or without 30 mg/mL of total tumor proteins extracted from LLC tumors 18 h after PTA or Sham. TGF-β and IL-4 in the cultivation condition were adjusted to 10 ng/mL and 20 ng/mL, respectively. In some experiments, IL-1R antagonist Anakinra (100 ng/mL), anti-IL-1β mAb (150 ng/mL), anti-TNF-R1 (50 ng/mL) plus anti-TNF-R2 mAbs (50 ng/mL), STAT1 phosphorylation inhibitor SU6656 (150 µM), or IRF1 expression inhibitor cyanidin 3-O-glucoside chloride (C3G, 300 µM) were added to investigate the signaling pathways that engaged in the PTA-induced Th9 cells differentiation.

For in vitro human Th9 cell polarization, the peripheral blood mononuclear cells (PBMCs) were isolated from NSCLC patients using 1×Ficoll and further purified by flow cytometry with the MojoSort Human CD4^+^ T Cell Isolation Kit (BioLegend). Then, the isolated CD4^+^ T cells were stimulated with plate-bound anti-human CD3ε (5 µg/mL) and anti-CD28 (5 µg/mL) antibodies and further differentiated into Th9 cells in T cell culture medium (RPMI 1640, 10% FBS, 100 U/mL penicillin, and 100 mg/mL streptomycin) supplemented with human IL-4 (20 ng/mL) and TGFβ1 (10 ng/mL).

### Mice tumor models

For the subcutaneous (*s.c.*) graft tumor model, LLC cells that grew at a logarithmic speed were *s.c.* injected into the flank of C57BL/6 mice aged 6–8 weeks (2 × 10^6^/mouse). The tumor volumes were evaluated every 2 days by using a vernier caliper. The equation applied to calculate the tumor volume was as follows: Volume = (major tumor axis) × (minor tumor axis)^2^ × π/6. For the recurrence model, LLC/LUC cells that grew at a logarithmic speed were *s.c.* implanted into the right flank of C57BL/6 mice (8 × 10^5^/mouse), the tumor growth was visualized by intraperitoneal injection of 100 mg/kg XenoLight D-Luciferin Potassium Salt and monitored by the PerkinElmer IVIS Spectrum (PerkinElmer, Waltham, MA, USA). The tumor and the adjacent tissues were radically resected when its volume reached 200–300 mm^3^. After recovering for 2–3 weeks, PerkinElmer In Vivo Imaging System (IVIS) Spectrum was used to check tumor recurrence, and mice with local recurrence were excluded to avoid surgery-induced bias. Then, the tumor-free mice were rechallenged by *s.c.* implantation of LLC/LUC cells (4 × 10^6^/mouse) at the left flank, followed by monitoring the tumor growth over time. For the metastasis model, C57BL/6 mice received intravenous (*i.v.*) injection of LLC/LUC cells (8 × 10^5^/mouse) and *s.c.* implantation of LLC cells (2 × 10^6^/mouse). The tumor metastasis was visualized by PerkinElmer IVIS Spectrum at the indicated days. Mice were treated and sacrificed on the indicated days, and the tumors and lungs were harvested and weighed. For some experiments, the tumor-infiltrating immune cells were isolated and further analyzed by flow cytometry.

The tumor tissues were digested with RPMI 1640 modified medium supplemented with 0.1 mg/mL DNase I and 0.5 mg/mL Collagenase II and IV, then straining through the 70 μm strainer to isolate single cells. Then, the tumor-infiltrating immune cells were isolated using 37% Percoll and assessed by flow cytometric analyses. The tumor-infiltrating CD45^+^CD4^+^ T cells were flow-cytometric sorted from the isolated immune cells and further detected for gene expression via qPCR analyses. Meanwhile, the peripheral blood from tumor model mice was also collected for ELISA tests to evaluate the cytokines.

### Percutaneous thermal ablation

MWA was selected as the PTA technique in our study and was performed using the MTC-3 C Microwave Ablation Machine (Vison-China Medical Devices R&D Center, Jiangsu, China) following the protocol previously described with minor revision [25]. Briefly, tumor-bearing mice received anesthesia with isoflurane inhalation and were fastened on the operating table. Then, the ablation area was disinfected with 75% ethanol and exposed by an approximately 0.5 cm incision, followed by a single PTA at 90–100 ℃ for 90–120 s. Then, the incision was closed with intermittent sutures. Mice merely underwent anesthesia and the sham surgery served as control. After the operation, mice were maintained on the electric blanket with a temperature of 37 ℃ until resuscitation.

### Adoptive Th9 cell transfer therapy

The adoptive Th9 cell transfer was performed as described previously [17, 21, 24]. Briefly, C57BL/6 mice (6–8 weeks of age) underwent the *i.v.* injection of Th9 cells (5 × 10^6^ cells/mouse) differentiated in vitro for 3–4 days on the indicated days after tumor inoculation, and mice treated with PBS were used as the control. The tumor growth and survival of mice were supervised over time. To treat the patient-derived xenografts (PDX) mice model, the in vitro differentiated NSCLC patient-derived Th9 cells (3–5 × 10^6^ cells/mouse) were *i.v.* administrated on the indicated days.

### H&E staining

The tumor and lung tissues were freshly harvested from mice and fixed with 4% neutral buffered formalin (Servicebio Inc., Wuhan, China) immediately. Subsequently, the tissues were embedded in paraffin and were sectioned with a thickness of 5 μm. The tissue sections were placed on the adhesion microscope slides (Citotest Scientific Co., Ltd., Jiangsu, China) and flowed by staining with H&E using the standard protocol described previously. Briefly, tissue sections were dewaxed and hydrated and then stained as follows: washing in flowing water, staining in Harris hematoxylin solution (Baso Diagnostics Inc., Guangdong, China) for 30 s, washing in flowing water, 1% acid alcohol for 3 s, washing in flowing water, staining in eosin for 30 s, followed by dehydration with 80%, 95%, and 100% ethanol and xylene for 5 min, respectively. Finally, the tissue sections with the H&E staining were sealed with coverslips and magnified with the microscope.

### Cytokine determination

Mice IL-1β, TNF-α, TGF-β, and IL-4 levels and human IL-1β and TNF-α levels were evaluated using single-plex sandwich enzyme-linked immunosorbent assay (ELISA) kits (Excel Biotech, Shanghai, China). Mice and human IL-9 levels were determined using a single-plex sandwich ELISA kit (Beyotime Biotech, Shanghai, China). The assay was conducted per the manufacturer’s instructions.

### Extraction of tumor proteins and measurement of cytokines

LLC tumors were dissected from mice and were digested with RIPA supplemented with protease and phosphatase inhibitor, phenylmethanesulfonyl fluoride (PMSF), and Aprotinin, followed by centrifuge at 12,000 rpm for 20 min at 4 ℃. Then, the lysates were collected and sterilized by filtering with 0.22 μm strainers. The cytokines levels were determined by enzyme-linked immunosorbent assay (ELISA) tests.

### Immune cell staining and flow cytometry

The immune cell staining and flow cytometry analysis were performed per the previously described methods [24]. For cell surface staining, cells were incubated with the indicated flow cytometric antibodies in the staining buffer (2.5% FBS in PBS) on ice for 25 min, followed by washing with the staining buffer at least twice. Then the cells were incubated with the Zombie UV Fixable Viability Kit and washed with the staining buffer, followed by the fixation by 4% paraformaldehyde and washing at least twice with the staining buffer. Finally, the cells were diluted in PBS and analyzed by the BD LSRFortessa Cell Analyzer (BD Biosciences). For intracellular cytokine staining, cells were stimulated for 8–16 h at 37 °C, 5% CO_2_ in the T cells culture medium (RPMI 1640, 10% FBS, 100 U/mL penicillin, 100 mg/mL streptomycin, and 1×levofloxacin) supplemented with the Leukocyte Activation Cocktail, with BD GolgiPlug (BD Pharmingen), followed by incubating with the Fixation/Permeabilization Buffer Solution (BD Biosciences) according to the protocol from the manufacturer. Subsequently, cells were incubated with the indicated intracellular cytokine flow cytometric antibodies in the staining buffer (2.5% FBS in PBS) on ice for 30 min, followed by washing with the staining buffer at least twice. Finally, the cells were diluted in PBS and analyzed by the BD LSRFortessa Cell Analyzer. The flow cytometric data were analyzed using Flowjo V10.

### RNA isolation and real-time quantitative PCR

The total RNA from in vitro differentiated or tumor-infiltering Th9 cells was extracted using TRIzol per the manufacturer’s protocol, followed by cDNA amplifying using the PrimeScript RT Reagent Kit (Takara). Real-time qPCR was carried out with SYBR Green Master Mix (Roche), and the expression of mice *Il-9*, *Pu.1*, *Irf1*, *Irf4*, *Stat1*, *Stat3*, *Stat5*, *Stat6*, *Traf6*, *Nf-κb1*, *Nf-κb2*, *Gata3*, *Map3k8*, *Eomes*, *Gzma*, *Gzmb*, *Gzmk*, and *Il1r1* genes and human *IL-9* and *IRF1* genes were measured by the standard curve method, followed by normalizing to the expression level of mice or human *Actb*. The qPCR primers applied in the study are listed in Table S1.

### Western blot assay

Total proteins were extracted using lysis buffer (RIPA), followed by measuring the protein concentration using Pierce BCA Protein Assay Kit (Thermo Fisher Scientific). The extracted proteins were then electrophoresed with sodium dodecyl sulfate-polyacrylamide gel electrophoresis at 80 V for 40 min, followed by electrophoresing at 100 V for 80 min. The BLUeye Prestained Protein Ladder (GeneDireX, Inc.) was used as the reference. Then, the separated proteins were transferred to the polyvinylidene fluoride membrane by using trans-blotting apparatus at 100 V for 120 min. The membrane containing the indicated protein was blocked with 5% NON-Fat Powdered Milk (Sangon Biotech Co. Ltd.) at 20–25 °C for 1 h. The blocked membrane was then incubated with the indicated primary antibody against IRF1, STAT1, p-STAT1, or β-actin (1:1000) at 4 ℃ for 14–16 h, followed by washing with PBST (PBS with 0.1% Tween) four times. Then, the membrane was incubated with the HRP-linked secondary antibodies (1:15000) at 20–25 °C for 2 h, followed by washing with PBST four times. The protein band was visualized with the Immobilon Western HRP Substrate Peroxide Solution (EMD Millipore Co., Burlington, MA, USA) using ChemiScope (ClinX Science Instruments, Shanghai, China). The expression level of the protein was assessed by analyzing the intensity of the indicated band with ImageJ (NIH Image, Bethesda, MD, USA).

### RNA-sequencing analysis

Mouse naive CD4^+^ T cells cultured under Th9-polarizing conditions for 72 h were applied for total RNA extraction with TRIzol (Invitrogen) and subjected to RNA sequencing analysis. RNA sequencing was performed by BGI Tech Solutions (Shenzhen, China). The raw transcriptomic reads were mapped to a reference genome (GRCm38/mm10) using Bowtie. Gene expression levels were quantified using the RSEM software package. Significantly affected genes were acquired by setting a fold change > 1.5 and a false discovery rate threshold of 0.05. Differentially expressed genes were analyzed using the Ingenuity Pathway Analysis and DAVID bioinformatics platforms.

### Mouse CD4^+^T cell *Il1r1* knockdown

The gene silencing in mouse CD4^+^ T cells followed a previously established protocol from our team [24]. To achieve *Il1r1* knockdown, retrovirus particles were produced by transfecting HEK293T cells with the MSCV-PIG plasmid, containing specific short hairpin RNA (shRNA) sequences, in conjunction with the envelope and packaging plasmids pCL-Eco, using Lipofectamine 2000. After 48 h, the supernatant, containing fresh retrovirus, was harvested and used to infect naive CD4^+^ T cells, previously stimulated with anti-CD3ε (5 µg/mL) and anti-CD28 (5 µg/mL) for 18–24 h. The infected T cells underwent centrifugation at 1800 g for 1.5 h in the presence of polybrene (8 µg/mL) and were then cultured at 37 °C for an additional 2–6 h before suspension in mouse Th9 cell differentiation medium. The integrity of the constructs was confirmed by DNA sequencing, and the efficiency of *Il1r1* knockdown was assessed using qPCR. The sequences of shRNA applied were as follows:

scrambled control shRNA,

5′-CCGGCCTAAGGTTAAGTCGCCCTCGCTCGAGCGAGGGCGACTTAACCTTAGGTTTTTG-3′;

*Il1r1*-shRNA,

5’-CCGGCGTGAGCTTCTTCGGAGTAAACTCGAG TTTACTCCGAAGAAGCTCACGTTTTTG-3’.

### Humanized patient-derived xenograft mice model

Tumor tissues from NSCLC patients who underwent surgery at Shanghai Lung Cancer Center, Shanghai Chest Hospital were *s.c.* implanted into immunodeficient NTG mice on day 0. Then, PBMCs isolated from NSCLC patients were deleted with CD4^+^ T cells and activated in vitro, followed by *i.v.* injected into NTG mice (8–10 × 10^6^ cells/mouse) on day 7. Sham or PTA was performed on day 10, and 18–24 h later, human Th0 or Th9 cells (4–5 × 10^6^ cells/mouse) were *i.v.* injected. On day 21, the PDX mice were sacrificed, and the NSCLC tumors were harvested and weighed, followed by the isolation of the infiltrated immune cells, which were further flow-cytometric analyzed or sorted for qPCR tests. Meanwhile, the peripheral blood of PDX mice was collected for ELISA tests to assess the cytokines.

### Human peripheral blood samples

Human peripheral blood was obtained from NSCLC patients aged 35–65 who underwent surgery at Shanghai Lung Cancer Center, Shanghai Chest Hospital. PBMCs were immediately isolated and cryopreserved at -80 ℃ using FBS supplemented with 10% dimethyl sulfoxide (Merck-Sigma Aldrich Co. LLC., St. Louis, MO, USA).

### Statistical analysis

The data are expressed as mean ± SEM. The presented data in the study are the representative results of at least three independent experiments unless indicated otherwise. As indicated, the statistics were analyzed by two-tailed Student’s t-test or one-way ANOVA with post hoc Tukey’s analysis. For Kaplan-Meier analysis of survival proportion, the death of tumor-bearing mice, the tumor volume reached 1800 mm^3^, or the major tumor axis reached 20 mm were considered the event. The statistical analysis was conducted using GraphPad Prism 9. The two-tailed *p*-value of < 0.05 was supposed to be statistically significant and was indicated by ^*^, those at the two-tailed *p*-value of < 0.01 were indicated by ^**^, and those at the two-tailed *p*-value of < 0.001 were indicated by ^***^.

## Results

### PTA, in combination with adoptive Th9 cell transfer therapy, exhibits robust therapeutic efficacies against NSCLC tumors

Previous studies have indicated PTA induces the release of pro-inflammatory cytokines and TSAs and further enhances the anti-tumor immune response [25, 26]. However, PTA also contributed to elevated PD-1 expressions and may be associated with tumor progression [27]. Therefore, we first investigated the therapeutic efficacy of PTA monotherapy against LLC tumors, revealing that PTA merely slightly delayed tumor growth and extended the survival of tumor-bearing mice, though potently caused coagulation necrosis in the primary tumor foci (Figure S1**A**-**F**). Then, we detected the impacts of PTA on the TME by applying flow cytometry following the gating strategy described in Figure S1 G, which, as expected, found that PTA increased the number of immune cells (CD45^+^ cells) in TME (Figure S1 H). Further analyses of the immune cell subpopulation showed that PTA simultaneously increased the absolute number of CD11b^+^ myeloid cells and pan-DCs (F4/80^−^ CD11c^+^ MHCII^+^ cells), but not lymphocytes (CD3^+^ CD11b^−^ cells), nature killer (NK) cells (CD3^−^ NK1.1^+^ cells), and pan-B cells (CD45R/B220^+^ cells). Furthermore, we detected a significantly raised number of M1-like Mφ (CD11b^+^ F4/80^+^ MHC II^+^ cells) and a decreased frequency of M2-like Mφ (CD11b^+^ F4/80^+^ CD206^+^ cells) in tumors from mice receiving PTA while observing no notable alteration in the number of polymorphonuclear MDSCs (PMN-MDSCs, CD11b^+^ F4/80^−^ CD11c^−^ Ly6G^+^ Ly6C^lo^ cells) or monocytic MDSCs (m-MDSCs, CD11b^+^ F4/80^−^ CD11c^−^ Ly6G^−^ Ly6C^hi^ cells; Figure S1 I-J). Moreover, we also noticed that despite PTA possessing restricted impacts on the number of tumor-infiltrating CD4^+^ T cells, Th1 (CD4^+^ IFN-γ^+^) cells, Th2 (CD4^+^ IL-4^+^) cells, and Tregs (CD4^+^ Foxp3^+^ CD25^+^ CD127^−^ cells), it significantly improved the infiltration of Th9 (CD4^+^ IL-9^+^) cells and CD8^+^ T cells into the tumor lesions, while it neither enhanced the expression of anti-tumor cytokines, including IFN-γ, granzyme B, and IL-9 nor attenuated the expression of PD-1 in CD8^+^ T cells (Figure S1 K-M). Taken together, these results suggested that PTA induced moderate and short-lived anti-tumor efficacies and exhibited limited abilities to generate a potent anti-tumor immune response but may contribute to the differentiation and IL-9 expression of Th9 cells in the primary tumor foci.

**Fig. 1:**
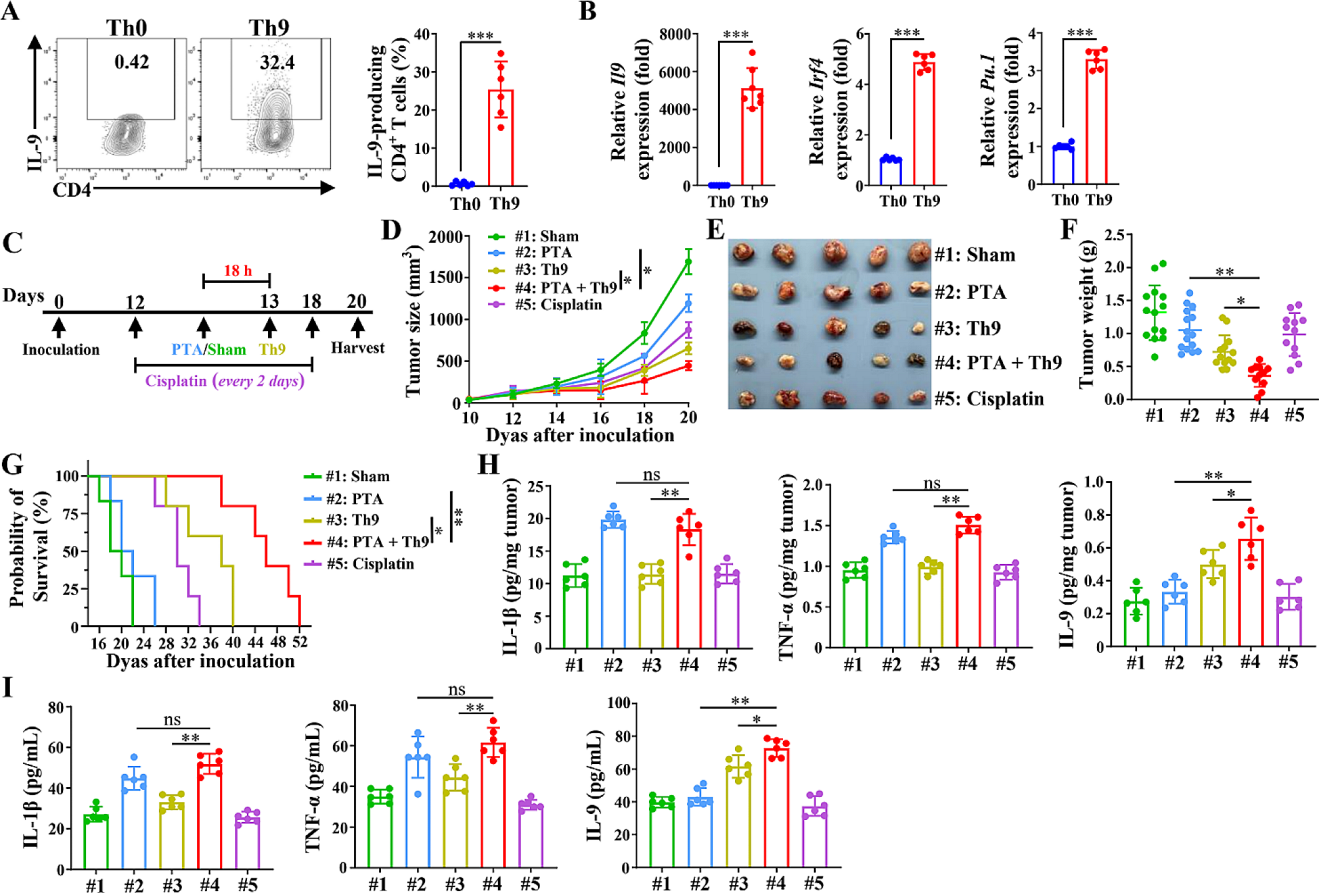
Combining PTA and adoptive Th9 cell transfer therapy induces notable anti-tumor effects. (**A**) Flow cytometric analysis of the frequencies of IL-9-producing CD4^+^ T cells under Th0 or Th9 differentiation conditions for 3–4 d (left) and the corresponding statistical analysis (right). (**B**) qPCR analysis of the mRNA expression of Th9 signature genes (left: *Il9*, middle: *Irf4*, and right: *Pu.1*) of CD4^+^ T cells under Th0 or Th9 differentiation conditions for 3–4 d. Results were normalized to the expression of *Actb* and are presented in relation to that of Th0 cells. (**C**) The timeline of treatment. C57BL/6 mice aged 6–8 weeks were *s.c.* inoculated with 2 × 10^6^ LLC cells. 12 days later, mice were randomly assigned to 5 groups (*n* = 14–16 mice/group) and received sham + PBS (Sham group), PTA + PBS (PTA group), sham + adoptive Th9 cell transfer (Th9 group), PTA + adoptive Th9 cell transfer (PTA + Th9 group), or sham + cisplatin (Cisplatin group), respectively. Mice were sacrificed on day 20. Tumor growth (**D**), representative tumor images (**E**), tumor weights (**F**), and Kaplan-Meier survival analysis (**G**) of C57BL/6 mice that underwent Sham, PTA, adoptive Th9 cell transfer, PTA plus adoptive Th9 cell transfer, or cisplatin (*n* = 14–16 mice/group), respectively. ELISA tests of IL-1β (left), TNF-α (middle), and IL-9 (right) levels in the tumor (**H**) and serum (**I**) in LLC-bearing mice on day 20, as described in (**C**). #1: Sham; #2: PTA alone; #3: Th9 alone; #4: PTA + Th9; #5: Cisplatin. Student’s t test or one-way ANOVA with Tukey’s post hoc analysis specified for #2 vs. #4 and #3 vs. #4 was used. Bars, mean; error bars, SD; ^*^, *p* < 0.05; ^**^, *p* < 0.01; ^***^, *p* < 0.001; and ns, not significant

Based on the aforementioned results, we sought to combine PTA with adoptive Th9 cell transfer therapy to access a more potent immunotherapy approach against NSCLC tumors. Firstly, mice Th9 cells that highly expressed Th9-related genes, including *Il9*, *Irf4*, and *Pu.1*, were generated by proliferating naïve CD4^+^ T cells in vitro for 3–4 days (Fig. 1A-B). Intriguingly, we found that the proportion of IL-9^+^ cells merely account for approximately 30% of the CD4^+^ cells, and thus, we then performed the detection om the IL-9^−^ subgroup. IL-9-reporter mice (expressing IL-9 linked with GFP) were employed, and GFP^+^ CD4^+^ T cells (representing Th9 cells) and GFP^−^ CD4^+^ T cells (comprising the remaining 70% of cells) were sorted using flow cytometry and subjected to bulk-RNA sequencing. Figure S2 A illustrates the top 25 differentially expressed genes between GFP^−^ and GFP^+^ cells. As anticipated, *Il9* emerged as the most significantly varied gene between the two cell populations. Furthermore, we found a significantly higher expression of *Il9* in GFP^+^ cells, while GFP^−^ cells exhibited notably elevated expression of genes related to effector T cell development (*Eomes*) and the granzyme panel (*Gzma* and *Gzmk*, Figure S2 B). Interestingly, GFP^−^ and GFP^+^ cells exhibited comparable expression levels of all selected genes that have been reported to be pivotal to Th9 cell differentiation, except for *Gata3*. Additionally, both cell types demonstrated similar expression levels of selected exhaustion/senescence and memory/stemness genes. Finally, we validated these results and further investigated the differences between Th0 and GFP^−^ cells. The comparison between the GFP^+^ and GFP^−^ cells demonstrated results aligned with that of the bulk-RNA sequence (Figure S2 C-D). Intriguingly, GFP^−^ cells showed a significantly elevated expression of the *Il9* gene along with *Pu.1* and *Irf4* genes (contribute to Th9 cell differentiation induced by IL-4 and TGF-β in our proliferation condition) and comparable *Gata3* expression compared with Th0 cells. Given this, we hypothesize that IL-9^−^ cells undergo a process of transitioning towards IL-9-producing cells under the Th9 proliferation condition with IL-4 and TGF-β, with the expression of the *Il9* gene being temporarily inhibited in vitro. Therefore, IL-9^−^ cells are likely associated with upregulated *Il9* expression and IL-9 production and exhibit Th9-like anti-tumour efficacy after being adoptively transferred into mice. Moreover, *Gata3* may play a pivotal role in *Il9* expression induced by IL-4 and TGF-β in vitro.

Then, we detected the dynamic change of IL-1β and TNF-α in the serum and tumor of mice within 72 h following PTA, revealing a dramatical increase of these two critical pro-inflammatory cytokines during the 18–48 h period after PTA (Figure S1 N). Intriguingly, a rising tendency in IL-9 level was also observed from 36 h after PTA in the tumor and serum, suggesting that inflammation storm induced by PTA may favor the production of IL-9. Given this, Th9 cells were adoptively transferred 18 h after PTA to meet the peak of inflammation storm, which might maximize their IL-9 production and thus exhibit the best efficacies.

As described in Fig. 1C, the tumor-bearing mice were randomly distributed into the Sham, PTA, Th9, and PTA + Th9 group, with the Cisplatin group as the positive treatment control. We found that mice undergoing PTA plus Th9 transfer therapy had significantly slower tumor growth and smaller tumor size and weight than those who received the monotherapy (Fig. 1D-F). Meanwhile, mice in the PTA + Th9 group also exhibited a notably longer survival duration than those in the monotherapy groups (Fig. 1G). Moreover, we detected IL-1β, TNF-α, and IL-9 levels in serum and tumor of tumor-bearing mice, which are essential for Th9 differentiation or anti-tumor efficacy. We found IL-1β and TNF-α levels were significantly increased by PTA alone and combinatorial therapy but not by adoptive Th9 cell transfer monotherapy, suggesting that PTA treatment rather than Th9 cell transfer influenced IL-1β and TNF-α secretion in tumor or plasma (Fig. 1H-I). Furtherly, the plasma and intertumoral IL-9 levels of the mice in the PTA + Th9 group were notably higher than that of the PTA and Th9 groups. These results suggested that the combination of PTA and adoptive Th9 cell transfer therapy induced robust therapeutic efficacy, superior to that of each monotherapy and cisplatin treatment against the established NSCLC tumor.

### PTA synergizes with adoptive Th9 cell transfer therapy to enhance the anti-tumor immune response of T cells

**Fig. 2:**
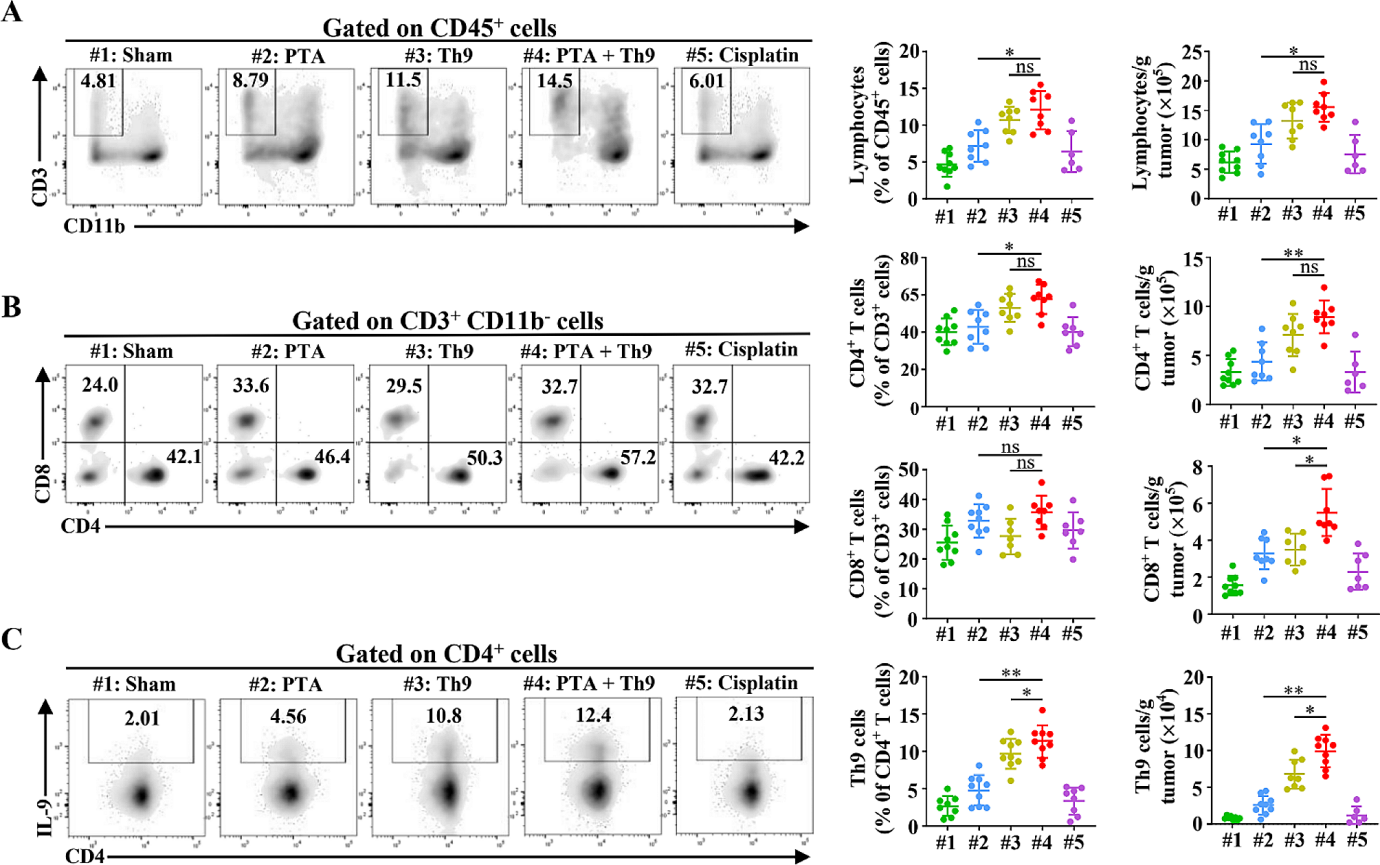
Combining PTA and adoptive Th9 cell transfer therapy synergistically increases tumor-infiltrating CD4^+^and CD8^+^T and Th9 cells. Flow cytometric analysis of tumor-infiltrating CD3^+^ CD11b^−^ lymphocytes (**A**), CD4^+^ and CD8^+^ T cells (**B**), and Th9 cells (**C**), as indicated in LLC-bearing mice on day 20, as described in Fig. 1**C**. Data are presented as representative plots (left) and summary graphs (right). #1: Sham; #2: PTA; #3: Th9; #4: PTA + Th9; #5: Cisplatin. Flow cytometric markers used to define immune cell subtypes (CD45^+^): lymphocytes, CD11b^−^ CD3^+^; CD4^+^ T, CD11b^−^ CD3^+^ CD8^−^ CD4^+^; CD8^+^ T, CD11b^−^ CD3^+^ CD4^−^ CD8^+^; Th9: CD4^+^ IL-9^+^. One-way ANOVA with Tukey’s post hoc analysis specified for #2 vs. #4 and #3 vs. #4 was used. ^*^, *p* < 0.05; ^**^, *p* < 0.01; and ns, not significant

We further detected the alteration of the tumor-infiltrating T cells in combinatorial therapy or monotherapy groups and found that combinatorial treated mice had a notably higher number of tumor-infiltrating T cells and CD4^+^ T cells than those that merely underwent PTA (Fig. 2A-B). Meanwhile, PTA, in combination with adoptive Th9 cell transfer therapy, also significantly increased CD8^+^ T cells in the TME compared with PTA or Th9 cell monotherapy (Fig. 2B). Furthermore, we analyzed the alteration in the subpopulation of CD4^+^ T cells, revealing that the combinatorial therapy remarkably increased the tumor-infiltrating Th9 cells while did not impact Th1 and Th2 cells in LLC tumors than PTA or adoptive Th9 cell transfer monotherapy (Fig. 2C, Figure S3**A**-**B**). These results suggested that PTA could synergize with adoptive Th9 cell transfer therapy to enhance the anti-tumor immune response of T cells.

### The combination of PTA and adoptive Th9 cell transfer therapy synergistically improves the anti-tumor effects of CD8^+^T cells

**Fig. 3:**
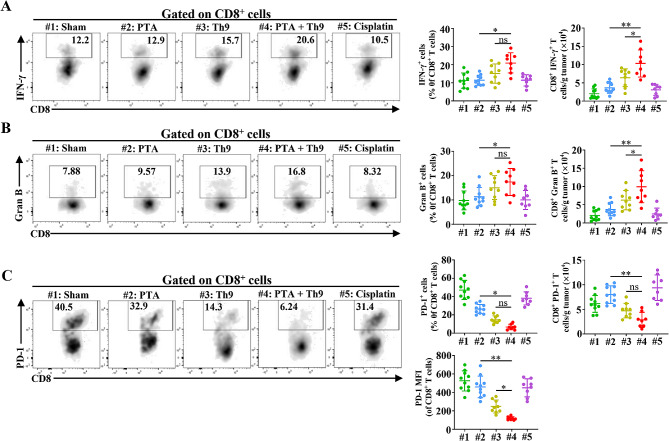
Combining PTA and adoptive Th9 cell transfer therapy synergistically enhances the anti-tumor effects of CD8^+^T cells. Flow cytometric analysis of tumor-infiltrating IFN-γ^+^ (**A**), Granzyme B^+^ (**B**), and PD-1^+^ (**C**) CD8^+^ (CD45^+^ CD11b^−^ CD3^+^ CD4^−^ CD8^+^) T cells and PD-1 MFI of CD8^+^ T cells, as indicated in LLC-bearing mice on day 20, as described in Fig. 1**C**. Data are presented as representative plots (left) and summary graphs (right). #1: Sham; #2: PTA; #3: Th9; #4: PTA + Th9; #5: Cisplatin. One-way ANOVA with Tukey’s post hoc analysis specified for #2 vs. #4 and #3 vs. #4 was used. ^*^, *p* < 0.05; ^**^, *p* < 0.01; and ns, not significant

CD8^+^ T cells are one of the most important cell types that induce anti-tumor immunity, and Th9 cells have been reported to activate CD8^+^ T cell responses and induce Tc9 cells (CD8^+^ IL-9^+^ cells), a subtype of CD8^+^ T cells that induces potent immune activity against malignancies [15, 28]. Therefore, we further evaluated the efficiency of CD8^+^ T cells in anti-tumor of combination or monotherapy groups. The results indicated that PTA, in combination with Th9 cell transfer, significantly enhanced the production of IFN-γ and granzyme B, two important anti-tumor cytokines, in CD8^+^ T cells compared with the monotherapy groups (Fig. 3A-B). Meanwhile, we also demonstrated that compared with PTA or adoptive Th9 cell transfer alone, combinatorial therapy possessed no significant impact on the tumor-infiltrating Tc9 cells (Figure S4) [28]. Upregulation of PD-1 could be implicated in the exhaustion of effector CD8^+^ T cells, and inhibition of PD-1 expression could enhance the anti-tumor efficacies of CD8^+^ T cells [29]. Therefore, we further evaluated the PD-1 expression of CD8^+^ T cells in all groups and found that although PTA slightly increased the absolute number of CD8^+^ PD-1^+^ cells compared with sham (not significant), the two kinds of treatments led to similar PD-1 expression levels in CD8^+^ T cells (Fig. 3C). Notably, the combinatorial therapy significantly reduced average PD-1 expression in tumor-infiltrating CD8^+^ T cells compared with PTA or Th9 cell monotherapy. Taken together, these results suggested that PTA and Th9 cells transfer synergistically promoted the anti-tumor immune responses of CD8^+^ T cells.

### PTA synergizes with adoptive Th9 cell transfer therapy to remodel tumor immunosuppressive microenvironments

**Fig. 4:**
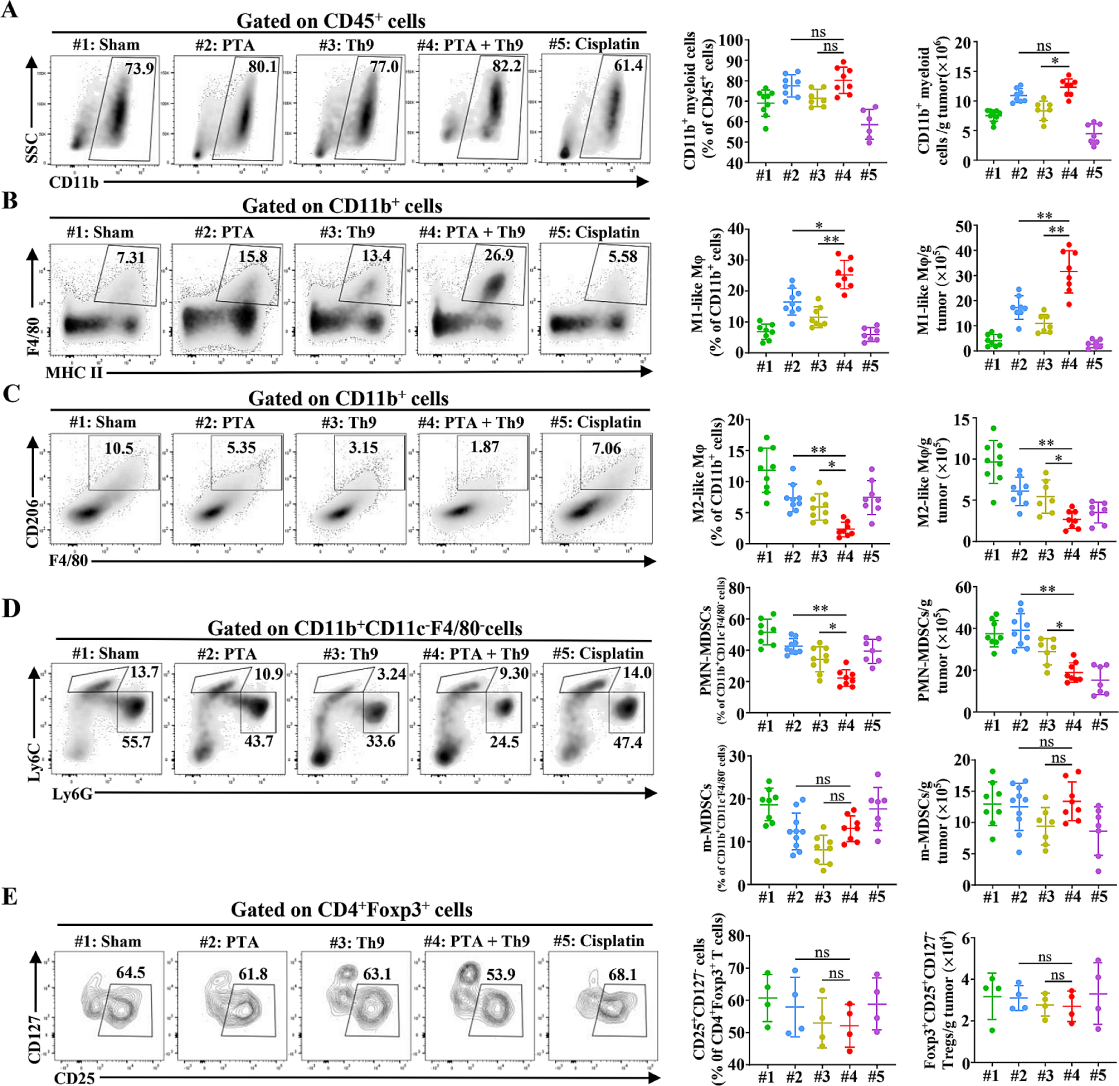
Combining PTA and adoptive Th9 cell transfer therapy synergistically remodels tumor immunosuppressive microenvironments. Flow cytometric analysis of tumor-infiltrating CD11b^+^ myeloid cells (**A**), M1-like (**B**) and M2-like (**C**) Mφ, PMN- and m-MDSCs (**D**), and Treg cells (**E**), as indicated in LLC-bearing mice on day 20, as described in Fig. 1**C**. Data are presented as representative plots (left) and summary graphs (right). #1: Sham; #2: PTA; #3: Th9; #4: PTA + Th9; #5: Cisplatin. Flow cytometric markers used to define immune cell subtypes (CD45^+^): M1-like Mφ, CD11b^+^ F4/80^+^ MHC II^+^; M2-like Mφ, CD11b^+^ F4/80^+^ CD206^+^; PMN-MDSCs, CD11b^+^ F4/80^−^ CD11c^−^ Ly6G^+^ Ly6C^lo^; m-MDSCs, CD11b^+^ F4/80^−^ CD11c^−^ Ly6G^−^ Ly6C^hi^; Tregs, CD11b^−^ CD3^+^ CD4^+^ Foxp3^+^ CD25^+^ CD127^−^. One-way ANOVA with Tukey’s post hoc analysis specified for #2 vs. #4 and #3 vs. #4 was used. ^*^, *p* < 0.05; ^**^, *p* < 0.01; and ns, not significant

TME is vital for anti-tumor immunity, tumor growth, and therapy resistance [30]. Given Figure S1 showed that PTA monotherapy could not effectively reverse the tumor immunosuppressive microenvironment, we then compared the difference of combinatorial therapy with monotherapies in remodeling the tumor immunosuppressive microenvironment, including the immunosuppressive myeloid cells-MDSCs, TAMs and Tregs, which could significantly impair cytotoxic CD8^+^ T cell-mediated anti-tumor immune responses and act as essential members of the immunosuppressive cells [31, 32]. The results indicated that CD11b^+^ myeloid cells in the TME of mice receiving combinatorial therapy were significantly more than that treated with adoptive Th9 cell transfer alone but similar to those that merely underwent PTA (Fig. 4A). Further analysis of cell subpopulation revealed that compared with PTA or adoptive Th9 cell transfer monotherapy, the combinatorial therapy notably decreased M2-like Mφ (also referred to as TAMs) in the TME while significantly increasing M1-like Mφ, which could promote anti-tumor immune responses of T cells via acting as antigen-presenting cells (APCs) and secreting proinflammatory cytokines (Fig. 4B-C). Additionally, PTA combined with adoptive Th9 cell transfer therapy dramatically reduced tumor-infiltrating PMN-MDSCs (the dominant cell subpopulation of MDSCs) than PTA alone (Fig. 4D). However, the combinatorial treatment did not significantly influence m-MDSCs and Tregs in the TME compared with the monotherapy, though a tendency toward lower IL-10^+^ Tregs was observed when comparing combinatorial therapy and monotherapy (Fig. 4D-E, Figure S5**A**-**C**). Finally, we also analyzed other substantial cell populations in TME, finding out that the combinatorial therapy could not notably impact pan-DCs, pan-B cells, and NK cells compared with PTA or adoptive Th9 cell transfer therapy alone (Figure S5**D**-**F**). These results suggested that PTA synergizes with adoptive Th9 cell transfer therapy to reverse tumor immunosuppressive microenvironments. We have also noticed that DCs are heterogeneous populations with anti-tumor and immunotolerant subgroups. Given we observed no significant alteration of the pan-DC groups when comparing combinatorial versus monotherapy, we did not further analyze the subgroup of pan-DCs. Further investigation may be necessary to detect the mechanism of activation of DCs and the alteration of their subgroups induced by PTA in promoting the anti-tumor efficacy of Th9 cells.

### PTA boosts Th9 cell-induced anti-tumor efficacy mainly via inducing IL-1β release

**Fig. 5:**
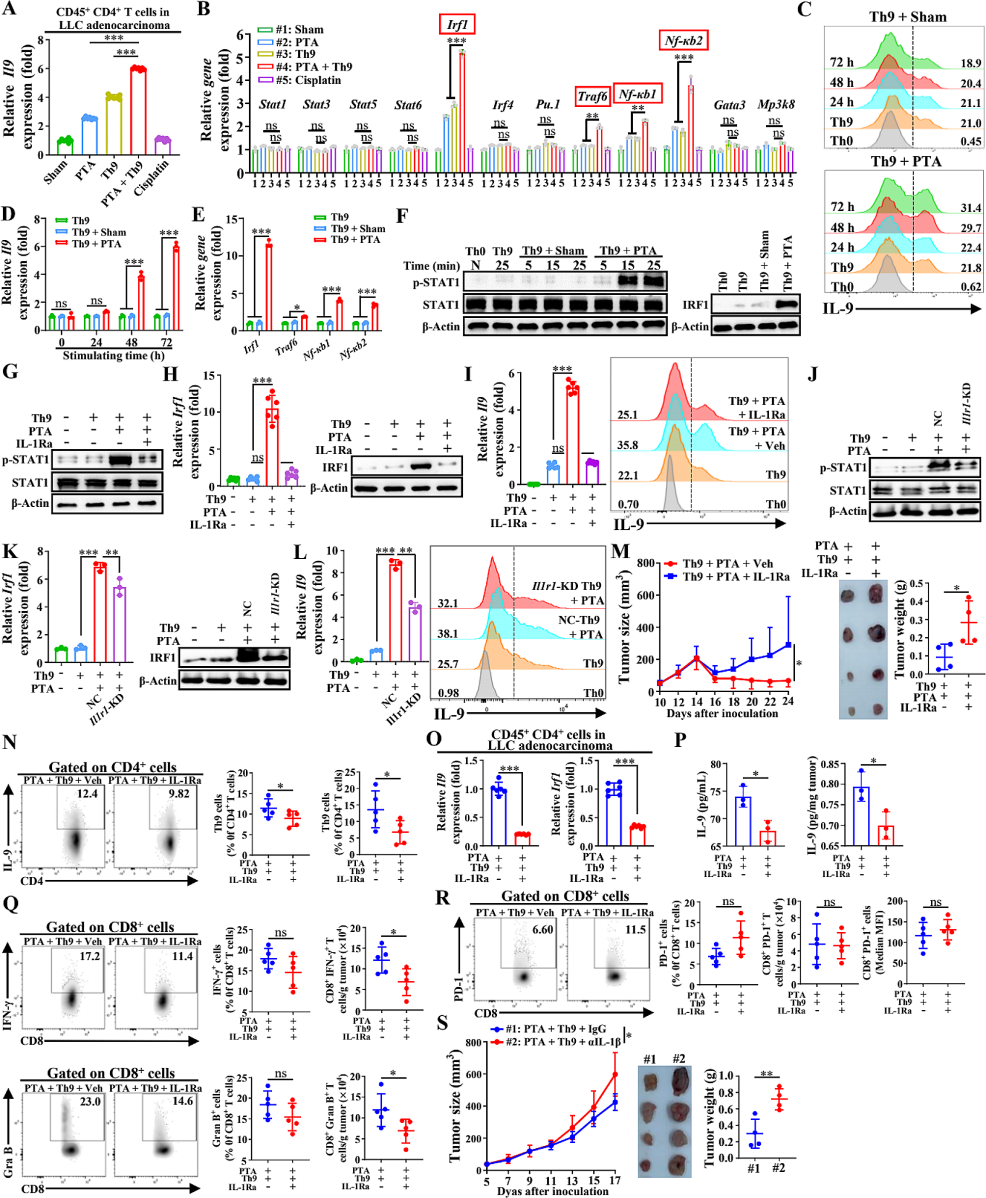
PTA promotes Th9 cell differentiation mainly via activating the IL-1β/STAT1/IRF1 pathway. qPCR analysis of *Il9* (**A**) and Th9-inducing gene (**B**) expression in tumor-infiltrating CD45^+^ CD4^+^ T cells isolated from LLC-bearing mice on day 20, as described in Fig. 1**C**. Results were normalized to the expression of *Actb* and are presented in relation to that of the Sham group. In (**C**-**I**), naïve CD4^+^ T cells were cultured under Th0 or Th9 conditions, with or without tumor lysate supernatants from 24 h-postoperative LLC-bearing mice underwent sham or PTA, and qPCR results were normalized to the expression of *Actb* and are presented in relation to that of control Th9 cells. Flow cytometric analysis of Th9 cells (**C**) and qPCR analysis of their *Il9* expression (**D**) cultured for 24, 48, or 72 h. (**E**) qPCR analysis of Th9-inducing genes in Th9 cells cultured for 72 h. (**F**) WB analysis of p-STAT and STAT in Th9 cells cultured for 5, 15, or 25 min (left), and IRF1 in Th9 cells cultured for 72 h (right). (**G**) WB analysis of p-STAT and STAT in Th9 cells cultured for 25 min, with or without IL-1Ra. qPCR (left) and WB (right) analysis of IRF1 expression (**H**), and qPCR (left) and flow cytometric (right) analysis of IL-9 expression in (**I**) Th9 cells cultured for 72 h, with or without IL-1Ra. (**J**) WB analysis of p-STAT and STAT in Th9 cells transfected with nonsense control shRNA (NC) or *Il1r1*-shRNA and cultured for 25 min. qPCR (left) and WB (right) analysis of IRF1 expression (**K**), and qPCR (left) and flow cytometric (right) analysis of IL-9 expression in (**L**) Th9 cells transfected with NC or *Il1r1*-shRNA and cultured for 72 h. (**M**) Tumor growth (left), representative tumor images (middle), and tumor weight (right) of C57BL/6 mice that revived the PTA plus adoptive Th9 cell transfer therapy, with or without IL-1Ra treatment (*n* = 6 mice/group). (**N**) Flow cytometric analysis of tumor-infiltrating Th9 cells, as indicated in LLC-bearing mice on day 24, as described in (**M**). Data are presented as representative plots (left) and summary graphs (right). (**O**) qPCR analysis of *Il9* (left) and *Irf1* (right) expressions in tumor-infiltrating CD45^+^ CD4^+^ T cells that were isolated from LLC-bearing mice on day 24, as described in (**M**), and then stimulated with anti-CD3 (5 µg/mL) and CD28 (5 µg/mL) for 24 h. Results were normalized to the expression of *Actb* and are presented in relation to that of the PTA + Th9 + Veh group. (**P**) ELISA tests of IL-9 levels in the serum (left) and tumor (right) in LLC-bearing mice on day 24, as described in (**M**). Flow cytometric analysis of tumor-infiltrating IFN-γ^+^ and Granzyme B^+^ (**Q**) CD8^+^ T cells and PD-1^+^ and PD-1 MFI of CD8^+^ T cells (**R**), as indicated in LLC-bearing mice on day 24, as described in (**M**). Data are presented as representative plots (left) and summary graphs (right). (**S**) Tumor growth (left), representative tumor images (middle), and tumor weight (right) of C57BL/6 mice that revived the PTA plus adoptive Th9 cell transfer therapy, with or without anti-IL-1β mAb (25 mg/kg/100 µL) treatment (*n* = 4 mice/group). One-way ANOVA with Tukey’s post hoc analysis or Student’s t test was used. Bars, mean; error bars, SD; ^*^, *p* < 0.05; ^**^, *p* < 0.01; ^***^, *p* < 0.001; and ns, not significant

Figure 1 has indicated that PTA could increase tumor-infiltrating Th9 cells and serum and intra-tumoral IL-9 levels and further significantly improve the anti-tumor efficacy of Th9 cells. Moreover, we isolated tumor-infiltrating CD45^+^ CD4^+^ T cells from LLC-bearing mice, as indicated in Fig. 1C, revealing that the combinatorial therapy notably up-regulated the expression of *Il9* gene compared with PTA or adoptive Th9 cell transfer monotherapy (Fig. 5A). Proinflammatory cytokines could enhance the anti-tumor effects of T cells, and many studies have combined T cell therapy with the administration of proinflammatory cytokines to improve immunotherapy [33]. Our previous results indicated that PTA could significantly increase serum and intra-tumoral IL-1β and TNF-α levels, which has been reported to enhance the differentiation of Th9 cells, secretion of IL-9, and anti-tumor efficacy against established solid tumors [21, 22]. Therefore, we conjectured that PTA might strengthen the ability of adoptively transferred Th9 cells against LLC tumors by inducing the release of proinflammatory cytokines. So, we first isolated tumor-infiltrating CD45^+^ CD4^+^ T cells from LLC-bearing mice and analyzed the expression of Th9-related genes, finding that compared with that in those treated with PTA or Th9 cell transfer alone, *Irf1, Traf6*, *Nfκb1*, and *Nfκb2* genes were notably upregulated in combinatorial treated mice (Fig. 5B). Meanwhile, we isolated lysates from LLC tumors 24–36 h after PTA or sham and detected the critical cytokines that related to Th9 cell differentiation (namely IL-1β, TNF-α, TGF-β, and IL-4) and IL-9 levels, and the results indicated that PTA notably increased IL-1β and TNF-α but not TGF-β, IL-4, and IL-9 levels compared to sham (Figure S6 A). The tumor lysates isolated from mice that received PTA, but not sham, significantly increased the proportion of Th9 cells and up-regulated expression of *Il9* gene when cocultured with naïve CD4^+^ T cells for 48–72 h under Th9 conditions (Fig. 5C-D). Accordingly, we then analyzed the expression of *Irf1, Traf6*, *Nfκb1*, and *Nfκb2* genes in naïve CD4^+^ T cells that proliferated with PTA or sham tumor lysates for 72 h, finding out that the PTA tumor lysates most highly up-regulated the *Irf1* gene among these four target genes compared with the sham tumor lysates (Fig. 5E). Most importantly, inhibiting IRF1 expression by cyanidin 3-O-glucoside chloride (C3G, a specific IRF1 inhibitor) effectively ameliorated PTA tumor lysates-induced *Il9* expression and Th9 cell proliferation in naïve CD4^+^ T cells (Figure S6**B**-**C**). STAT1 could act as a transcriptional activator of *Irf1* in response to proinflammatory cytokines, and phosphorylation of STAT1 at Tyr701 promotes the dimerization and subsequent translocation to the nucleus of STAT1 and is thus required for the transcriptional activity of STAT1 [22, 34, 35]. We found that in naïve CD4^+^ T cells, PTA tumor lysates time-dependently induced the phosphorylation of STAT1 at Tyr701 within 25 min of the induction of proliferation without altering the expression of STAT1 and increased downstream IRF1 expression when proliferated for 3 d (Fig. 5F). Nevertheless, disruption of STAT1 phosphorylation by SU6656 (a specific STAT1 phosphorylation inhibitor) notably inhibited IRF1 and *Il9* expression and Th9 cell differentiation in naïve CD4^+^ cells induced by PTA tumor lysates (Figure S6**D**-**F**). These results suggested that STAT1/IRF1 pathway is responsible for PTA tumor lysates-induced IL-9 expression and Th9 cell proliferation in vitro.

IL-1β is the dominant proinflammatory cytokine in the TME and the critical molecule that induces STAT1 phosphorylation and downstream *Irf1* expression in Th9 cells [22, 36]. Therefore, we conjectured that IL-1β played a pivotal role in the PTA-promoted Th9 cell differentiation. So, we disrupted the IL-1β signaling by a specific IL-1R antagonist (IL-1Ra)-Anakinra, and found that Anakinra effectively inhibited STAT1 phosphorylation and IRF1 expression induced by PTA tumor lysates (Fig. 5G-H). Accordingly, Anakinra inhibited PTA tumor lysates-induced *Il9* expression and Th9 cell differentiation (Fig. 5I). Considering the potential off-target effects of IL-1Ra, we then constructed *Il1r1*-KD CD4^+^ T cells and validated these results in vitro (Figure S6 G, Fig. 5J-L). Moreover, we applied neutralizing IL-1β (αIL-1β) mAb in PTA-induced Th9 cell proliferation condition in vitro, also demonstrating similar results (Figure S6**H**-**J**). Since TNF-α has been shown to activate an IRF1-dependent autocrine loop and STAT1-dependent signaling, we then investigate whether these phenotypes were specific to the IL-1β signaling [37]. The results showed that blocking TNFR (αTNFR) mAbs neither altered STAT1/IRF1 pathway nor affected IL-9 expression in PTA-induced Th9 cells (Figure S6**K**-**M**). Taken together, these results showed that PTA tumor lysates enhanced *Il9* expression and Th9 cell differentiation in vitro via the IL-1β/STAT1/IRF1 signaling pathway.

Furthermore, we ought to detect whether IL-1β plays a central role in the enhanced secretion of IL-9 and increased IL-9-producing CD4^+^ T cells in TME induced by PTA in vivo. Firstly, we treated LLC-bearing mice with PTA with or without IL-1Ra Anakinra and found that Anakinra did not significantly alter the impact of PTA on tumor growth (Figure S6 N). Additionally, flow cytometry analysis showed that Anakinra notably inhibited the induction of IL-9-producing CD4^+^ T cells in the TME by PTA while possessing limited effects on other populations of primary immune cells in the TME of PTA-treated LLC tumor (Figure S6 O). Moreover, Anakinra also intercepted the up-regulation of *Il9* and *Irf1* genes induced by PTA in the tumor-infiltrating CD4^+^ T cells but did not notably influence the serum and tumor IL-9 levels (Figure S6**P**-**Q**). These results indicated that IL-1β is pivotal in the PTA-induced proliferation of Th9 cells in vivo. However, the therapeutic efficacy of PTA against LLC tumors is quite limited, and thus, interference of IL-1β signaling could not notably impact the tumor growth in the NSCLC mice treated with PTA alone. Given this, we further assessed the impact of blocking IL-1β on the amplified anti-tumor effects of combinatorial therapy. The results showed that Anakinra did notably blockade the combinatorial therapy-induced efficacies to restrict LLC tumor development, magnify tumor-infiltrating Th9 cells, up-regulate *Il9* and *Irf1* expression in tumor-infiltrating CD4^+^ T cells, and evaluate serum and tumor IL-9 levels (Fig. 5M-P). Additionally, flow cytometric analysis revealed that Anakinra also significantly blocked the combinatorial therapy-induced reinforcement of anti-tumor effects of tumor-infiltrating CD8^+^ T cells and reversing of tumor immunosuppressive microenvironments, though it did not notably impact the absolute number of CD11b^+^ myeloid cells, DCs, and MDSCs (Fig. 5Q-R, Figure S6**R**-**T**). Finally, we confirmed these in vivo results by applying IL-1β mAb in mice, observing that IL-1β mAb notably compromised the anti-tumor efficacy of combinatorial therapy (Fig. 5S). Further analysis confirmed that IL-1β mAb effectively blocked PTA-induced promotion of Th9 cells and attenuated the synergistic effects of combinatorial therapy on the IFN-γ and granzyme B production in tumor-infiltrating CD8^+^ T cells (Figure S6**U**-**W**). Collectively, these results suggested that PTA induced Th9 cell differentiation and IL-9 production via increasing the IL-1β level in LLC-bearing mice and thus enhanced the anti-tumor efficacy of adoptive Th9 cell transfer therapy. Moreover, we also found that PTA increased the tumor-infiltrating DCs number and their expression of CD86, an activation marker of DCs (Figure S6**X**-**Y**). The maturity and activation of DCs could promote Th9 cell differentiation and its IL-9 production and cytotoxic effects [38–40].

Taken together, these results indicated that PTA enhanced the anti-tumor efficacy of tumor-infiltrating Th9 cells mainly by increasing IL-1β levels and further activating the downstream STAT1/IRF1 pathway. Meanwhile, the activation of tumor-infiltrating DCs may also contribute to the PTA-induced reinforcement of Th9 cell anti-tumor effects.

### PTA, in combination with adoptive Th9 cell transfer therapy, induces notable anti-tumor efficacies against PDX-NSCLC tumors

**Fig. 6:**
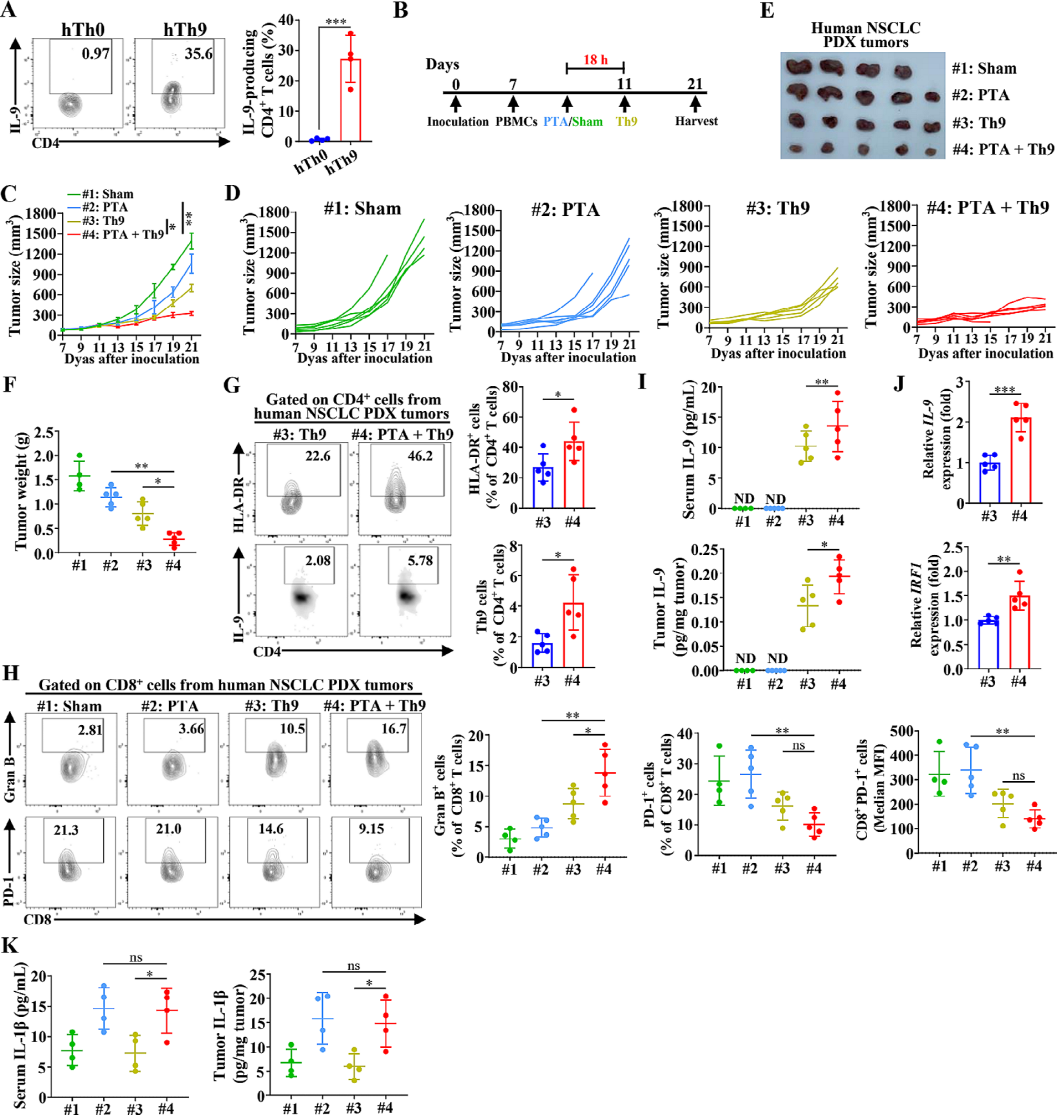
Combining PTA and adoptive Th9 cell transfer therapy significantly suppresses NSCLC PDX tumors. (**A**) Flow cytometric analysis of the frequencies of human IL-9-producing CD4^+^ T cells under Th0 or Th9 differentiation conditions for 4–5 d (left) and the corresponding statistical analysis (right). (**B**) The timeline of treatment. NTG mice aged 6–8 weeks were *s.c.* implanted with the tumor tissues from NSCLC patients. 7 days later, NTG mice were administrated with patients’ CD4^+^ T cell-depleted PBMCs and were randomly assigned to 4 groups (*n* = 6 mice/group), followed by receiving sham + Th0, PTA + Th0, sham + adoptive Th9 cell transfer, or PTA + adoptive Th9 cell transfer, respectively. Mice were sacrificed on 21. For each independent experiments, the tumor tissues were derived from the same patient to maintain the consistency of tumors among groups. (**C**) Summary graph of tumor growth. (**D**) Individual tumor growth of each mouse, and each line represents one mouse. (**E**) Representative tumor images. (**F**) Tumor weights. Flow cytometric analysis of the frequencies of tumor-infiltrating CD4^+^ HLA-DR^+^ and Th9 cells (**G**) and Granzyme B^+^ and PD-1^+^ and PD-1 MFI of CD8^+^ T cells (**H**), as indicated in human NSCLC PDX tumor-bearing mice on day 21, as described in (**B**). Data are presented as representative plots (left) and summary graphs (right). (**I**) ELISA detection of serum (upper) and tumor (lower) human IL-9 levels, as indicated in human NSCLC PDX tumor-bearing mice on day 21, as described in (**B**). (**J**) qPCR analysis of IL-9 (upper) and IRF1 lower) expression in tumor-infiltrating CD45^+^ CD4^+^ T cells isolated from human NSCLC PDX tumor-bearing mice on day 21, as described in (**B**), and then stimulated with anti-CD3 (5 µg/mL) and CD28 (5 µg/mL) for 24 h. Results were normalized to the expression of *ACTB* and are presented in relation to that of the #3 group. (**K**) ELISA detection of serum (left) and tumor (right) human IL-1β (**I**), as indicated in human NSCLC PDX tumor-bearing mice on day 21, as described in (**B**). #1: Sham + Th0; #2: PTA + Th0; #3: Sham + Th9; #4: PTA + Th9. One-way ANOVA with Tukey’s post hoc analysis specified for #2 vs. #4 and #3 vs. #4 or Student’s t test was used. Bars, mean; error bars, SD; ^*^, *p* < 0.05; ^**^, *p* < 0.01; ^***^, *p* < 0.001; and ns, not significant

Further, we detected the therapeutic efficacy of the combinatorial therapy against humanized patient-derived xenograft (PDX) mice tumors, aiming to evaluate its translational potential and clinical significance. Nonobese diabetic/ShiLtJGpt-*Prkdc*^em26Cd52^*Il2rg*^em26Cd22^/Gpt (NTG) mice were *s.c.* inoculated with human NSCLC tumor tissues and *i.v.* injected with CD4^+^ T cells-depleted PBMCs isolated from NSCLC patients to establish the humanized immune system. Human Th9 cells were generated by proliferating human CD4^+^ T cells in vitro for 4–5 days (Fig. 6A). Humanized PDX mice underwent sham surgery, PTA or adoptive human Th9 cell transfer alone or in combination (Fig. 6B). The results showed that PTA slightly delayed tumor growth compared with sham surgery control, while adoptive Th9 cell transfer induced a more significant therapeutic efficacy (Fig. 6C-F). Most importantly, PTA combined with adoptive Th9 cell transfer significantly suppressed tumor growth. The tumor volumes and weights of the mice receiving combined therapy were approximately half those adoptively transferred with human Th9 cells alone. Furthermore, flow cytometric analysis showed that combining PTA and adoptive Th9 cell transfer significantly increased tumor-infiltrating HLA-DR^+^ CD4^+^ T cells (the activated CD4^+^ T cells) and Th9 cells compared with adoptively transferred Th9 cells alone in humanized PDX mice (Fig. 6G). Meanwhile, combinatorial therapy also notably enhanced the granzyme B expression and suppressed PD-1 expression of CD8^+^ T cells in the TME of PDX mice compared with PTA or Th9 cell therapy alone (Fig. 6H). Accordingly, the serum and tumor IL-9 levels in the PDX mice undergoing the combinatorial therapy were notably higher than in those receiving PTA or Th9 cells monotherapy (Fig. 6I). Furthermore, PTA also remarkably promoted the expression of Th9-related genes, including *IL-9* and *IRF1*, in tumor-infiltrated CD4^+^ T cells in NSCLC PDX model, which was in line with the findings in NSCLC model using C57BL/6 mice (Fig. 6J). Finally, PTA alone or combined with adoptive Th9 cell transfer significantly increased IL-1β levels in the serum and tumor compared with transferring Th9 cells alone (Fig. 6K). These results confirmed that PTA could increase IL-1β and further promote the infiltration of human Th9 cells in TME, and most importantly, the combination of PTA and adoptive Th9 cell transfer therapy possesses robust efficacies and excellent therapeutic potential for the treatment of human NSCLC tumors.

### PTA, in combination with adoptive Th9 cell transfer therapy, effectively suppresses the recurrence of NSCLC tumors

**Fig. 7:**
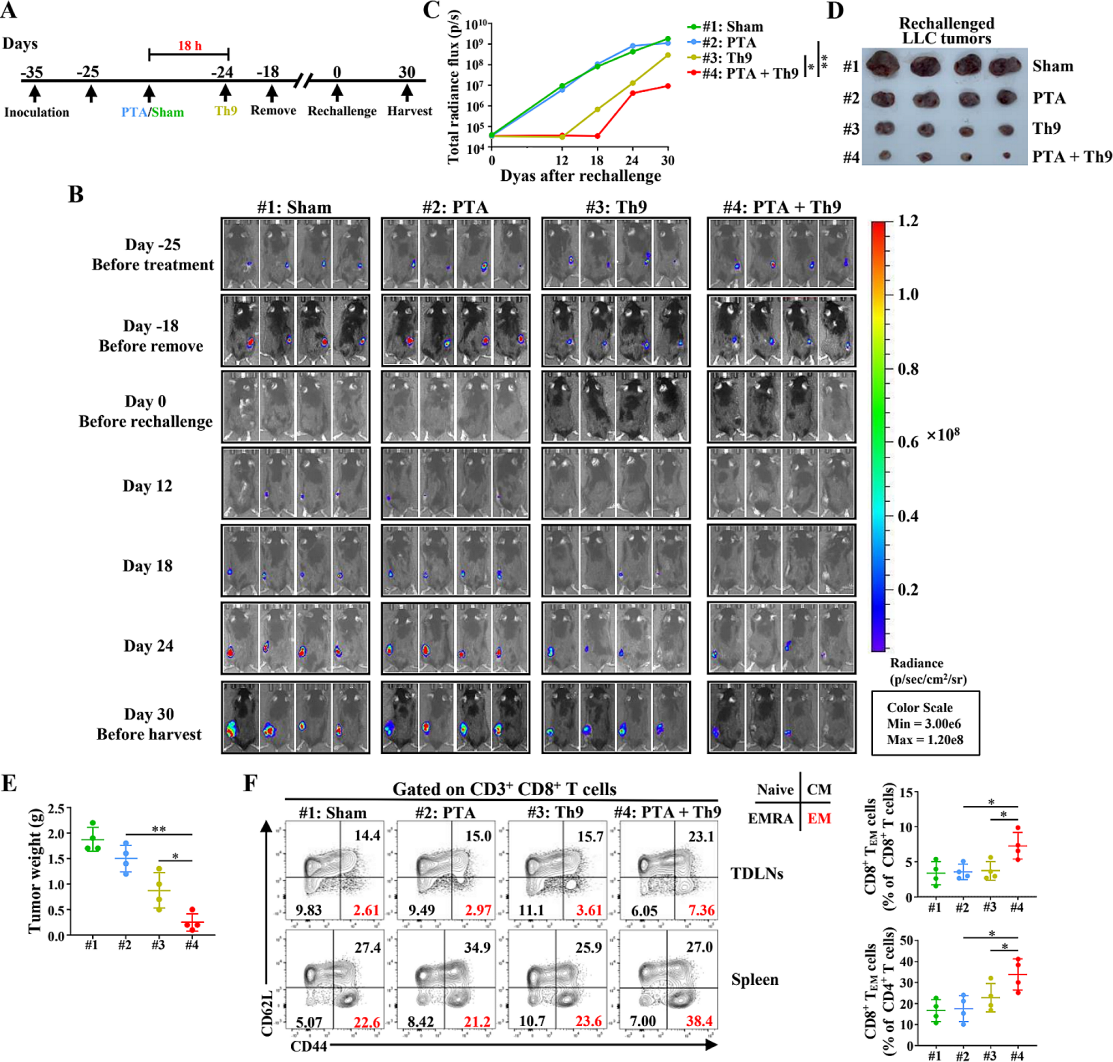
Combining PTA and adoptive Th9 cell transfer therapy notably suppresses the recurrence of LLC tumors. (**A**) The timeline of treatment. C57BL/6 mice aged 6 weeks were *s.c.* inoculated with 8 × 10^5^ LLC/LUC cells. 10 days later, mice were randomly assigned to 4 groups (*n* = 8 mice/group) and received Sham + PBS, PTA + PBS, Sham + adoptive Th9 cell transfer, or PTA + adoptive Th9 cell transfer, respectively. Mice were rechallenged with LLC/LUC tumors 2–3 weeks after surgical resections and were sacrificed on day 30. (**B**) Representative bioluminescence images of tumor growth over time. The tumor growth was monitored before the treatment (Day − 25), surgical resection (Day − 18), and rechallenge (Day 0) and 12, 18, 24, and 30 d after the rechallenge, respectively. (**C**) Tumor burden quantified as the total photon count from luciferase intensity of mice by IVIS imaging, representative tumor images (**D**) and rechallenged tumor weights (**E**). (**F**) Flow cytometric analysis of CD8^+^ T_EM_ cells in the TDLNs and spleen, as indicated in LLC/LUC-bearing mice on day 30, as described in (**B**). Data are presented as representative plots (left) and summary graphs (right). #1: Sham; #2: PTA; #3: Th9; #4: PTA + Th9. Flow cytometry markers used to define CD8^+^ T cell subtypes (CD45^+^ CD11b^−^ CD3^+^ CD8^+^): naïve T, CD44^lo^ CD62L^hi^; T_CM_, CD44^hi^ CD62L^hi^; T_EMRA_, CD44^lo^ CD62L^lo^; T_EM_, CD44^hi^ CD62L^lo^. One-way ANOVA with Tukey’s post hoc analysis specified for #2 vs. #4 and #3 vs. #4 was used. ^*^, *p* < 0.05; and ^**^, *p* < 0.01

Generally, tumor recurrence represents a failure in tumor immunotherapy and leads to a poor prognosis. Th9 cells represent a less-exhausted and hyperproliferative subset of CD4^+^ T cells, which exhibit a durable immune response against advanced tumors and inhibit tumor recurrence [17, 40]. Given this, we ought to investigate the potential efficacy of combinatorial therapy in preventing the recurrence of NSCLC using mice models with rechallenged LLC/LUC tumors (Fig. 7A). The results showed that combinatorial therapy dramatically inhibited the growth of rechallenged tumors compared with PTA or Th9 cell monotherapy (Fig. 7B-E). Furthermore, we detected the impacts of our treatment on the memory CD8^+^ and CD4^+^ T cells, which play a primary role in preventing tumor recurrence and are associated with the prognosis of NSCLC patients [41]. The results indicated that PTA combined with adoptive Th9 cell transfer dramatically increased the proportion of CD8^+^ T effector memory (CD8^+^ CD44^hi^ CD62L^−^, T_EM_) cells in the tumor drainage lymph nodes (TDLNs) and spleen of mice with tumor recurrence than PTA or Th9 monotherapy (Fig. 7F). Nevertheless, neither CD8^+^ T central memory (CD8^+^ CD44^hi^ CD62L^+^, T_CM_) nor CD8^+^ T_EMRA_ (CD8^+^ CD44^int/low^ CD62L^−^) cells were significantly altered by combinatorial therapy compared with monotherapy (Fig. 7F, Figure S7 A). Additionally, no remarkable difference was found among the mice receiving PTA and Th9 mono or combinatorial therapy concerning the proportion of CD4^+^ T_EM_, T_CM_, or T_EMRA_ cells in the TDLNs and spleen (Figure S7 B). Finally, the combinatorial treatment did not notably alter the proportion and the absolute number of tumor-infiltrating CD4^+^ or CD8^+^ T_RM_ (CD69^+^ CD103^+^) cells compared with PTA or Th9 treatment alone (Figure S7 C). Taken together, PTA plus adoptive Th9 cell transfer therapy increased the regional (TDLNs) and peripheral (spleen) CD8^+^ T_EM_ cells and effectively inhibited the recurrence of NSCLC tumors.

### PTA, in combination with adoptive Th9 cell transfer therapy, effectively suppresses lung metastasis of NSCLC tumors

**Fig. 8:**
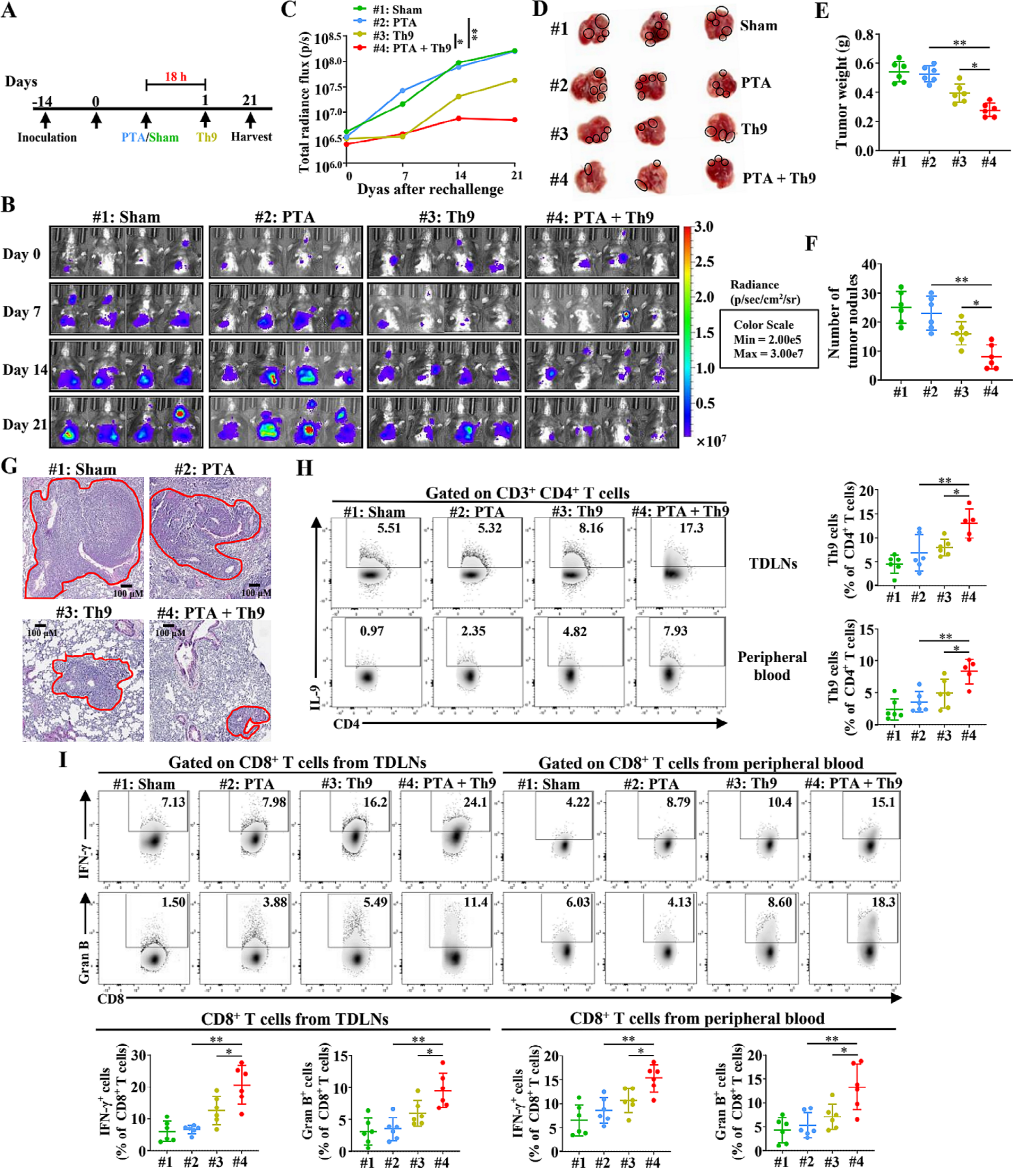
Combining PTA and adoptive Th9 cell transfer therapy effectively inhibits the lung metastasis of LLC tumors.**(A**) The timeline of treatment. C57BL/6 mice aged 6–8 weeks were *s.c.* and *i.v.* inoculated with 2 × 10^6^ LLC and 8 × 10^5^ LLC/LUC cells. 14 days later, mice were randomly assigned to 4 groups (*n* = 6 mice/group) and received Sham, PTA, adoptive Th9 cell transfer, or PTA plus adoptive Th9 cell transfer, respectively. Mice were sacrificed on day 21. (**B**) Representative bioluminescence images of lung-metastatic tumor growth over time. The tumor was monitored once a week. (**C**) The burdens of lung-metastatic tumors were quantified as the total photon count from luciferase intensity of mice by IVIS imaging. Representative images (**D**), weight (**E**), and statistical analysis (**F**) of the metastatic lung tumor foci of mice on day 21, as described in (**B**). (**G**) Representative H&E staining images of lung metastatic tumor foci of mice on day 21, as described in (**B**). The tumor area was marked with red lines. Flow cytometric analysis of Th9 cells (**H**) and IFN-γ^+^ and Granzyme B^+^ CD8^+^ T cells (**I**) in the TDLNs and peripheral blood, as indicated in tumor-bearing mice on day 21, as described in (**B**). Data are presented as representative plots (left/upper) and summary graphs (right/lower). #1: Sham; #2: PTA; #3: Th9; #4: PTA + Th9. One-way ANOVA with Tukey’s post hoc analysis specified for #2 vs. #4 and #3 vs. #4 was used. ^*^, *p* < 0.05; and ^**^, *p* < 0.01

Metastasis is the primary cause of cancer-related mortality and the most significant challenge in cancer treatment. Th9 cells have been found to possess powerful capabilities to inhibit lung metastasis of solid tumors, and thus, we detected whether the combinatorial therapy exhibited enhanced efficacy against lung metastasis of NSCLC tumors [14, 24]. C57BL/6 mice with local and metastatic NSCLC tumors underwent sham surgery, PTA or Th9 cell mono or combinatorial therapy (Fig. 8A). The results showed that combining PTA and adoptive Th9 cell transfer therapy dramatically suppressed lung metastasis of *i.v.* injected LLC/LUC cells compared with PTA or Th9 cell monotherapy (Fig. 8B-G). We subsequently used flow cytometry analysis to determine the contribution of CD4^+^ and CD8^+^ T cells in TDLNs and peripheral blood in generating systemic tumor metastasis control. As expected, combinatorial therapy significantly increased the proportion of Th9 cells in TDLNs and peripheral blood compared with monotherapy (Fig. 8H). Additionally, we observed that the percentage of INF-γ- and Granzyme B-producing CD8^+^ T cells in TDLNs and peripheral blood in the PTA + Th9 group was notably higher than in the PTA or Th9 group (Fig. 8I). Nevertheless, any treatment did not remarkably alter Th1 and CD8^+^ PD-1^+^ cells (Figure S8 A-B). These results indicated that the combinatorial therapy effectively enhanced anti-tumor efficacies of Th9 and CD8^+^ T cells in TDLNs and peripheral blood and further suppressed the lung metastasis of NSCLC tumors.

## Discussion

NSCLC remains one of the most prevalent malignancies worldwide, severely threatening global public health. Although immune checkpoint inhibitors have illuminated potential treatment avenues, elicit responses in only 20% of patients and carry the risk of adverse treatment-related events, including mortalities. PTA is a widely clinically applied minimally invasive technic for NSCLC, offering numerous advantages over surgical resection. However, PTA often incompletely eradicates tumor lesions, resulting in recurrence or progression shortly following treatment. Therefore, a novel therapeutic strategy with robust anti-tumor efficacy is urgently needed. In this study, we combined PTA with adoptive Th9 cell transfer therapy, revealing that combinatorial treatment displayed a more potent efficacy than PTA or Th9 cell monotherapy to inhibit NSCLC tumor growth, recurrence, and metastasis and prolong the survival of NSCLC model mice. Analysis of TME indicated that combinatorial therapy dramatically increased tumor-infiltrating Th9 cells, enhanced anti-tumor effects of CD8^+^ T cells, and remodeled tumor immunosuppressive microenvironments. Moreover, combinatorial therapy significantly strengthened the systemic immune response of CD8^+^ T cells in mice with tumor lung metastasis and induced peripheral CD8^+^ memory T cells in mice with tumor recurrence. Further mechanical study revealed that PTA enhances the proliferation and IL-9 production in Th9 cells and reinforces their anti-tumor efficacy primarily by upregulating interleukin-1β, subsequently activating the downstream STAT1/IRF1 pathway, which can be effectively blocked by intercepting IL-1β signaling. Finally, the enhanced ability of combinatorial therapy was further validated in the humanized PDX model. Hence, our research indicates that the combination of PTA and adoptive Th9 cell transfer therapy exhibits a robust and durable anti-tumor efficacy, emerging as a promising approach for treating NSCLC.

In the clinical treatment of NSCLC, the most commonly utilized PTA techniques include MWA, RFA), and cryoablation. For this study, MWA was chosen as the representative PTA method to integrate with adoptive Th9 cell transfer therapy [42]. In general, these thermal-based ablation techniques exert their tumor-eliminating effects by subjecting the targeted area to extreme temperatures, leading to tumor apoptosis and coagulative necrosis. Nevertheless, previous studies have highlighted several advantages of MWA over the other two methods in treating NSCLC patients. Both MWA and RFA employ electromagnetic waves to generate heat, inducing hyperthermic injury. However, unlike RFA, MWA does not rely on electric currents and tissue conduction, ensuring that its therapeutic delivery remains unaffected by desiccation [43]. Given this, MWA is better suited for lung tissues with high impedance compared to RFA [12]. Additionally, the microwaves utilized in MWA carry significantly higher energy and conduct faster and more efficiently, thus achieving better damage proposed for larger tumors and displaying lower susceptibility to heat-sink effects in comparison to RFA [12, 44]. In contrast to MWA, cryoablation eliminates tumor cells by introducing extremely low temperatures, as low as -160 ℃. Despite its potential to preserve more TSAs recognizable by the human immune system, cryoablation carries the risk of causing bleeding and thus is not suitable for treating tumors close to vessels [12, 45]. Given the abundant blood flow within the lung, the application of cryoablation is considerably restricted. Moreover, cryoablation does not lead to inflammation, a factor directly contributing to the proliferation and anti-tumor effects of Th9 cells. Based on these results, we deduce that MWA emerges as the most promising technology, offering superior efficacy and safety when integrated with Th9 cell-based immunotherapy. Additional research is imperative to pinpoint the optimal PTA approach for enhancing the anti-tumor efficacy of adoptive Th9 cell transfer therapy against NSCLC.

It has been widely accepted that PTA could directly damage tumor cells and lead to TSA release, which in turn, triggers an immune response against the tumors. Consistent with this consensus, our results also showed that PTA alone could increase the tumor-infiltrating total and mature DCs. The mature DCs express numerous co-stimulated molecules, including OX40L, CD80, CD86, and FASL, possessing the capability to induce Th9 cell differentiation and IL-9 production via various signaling pathways [46]. Additionally, our study found a substantial increase in tumor-infiltrating M1-like Mφ that devours TSAs and possesses the antigen-present capacity and thus functions as an essential subtype of APCs following PTA treatment [47]. Given this, the increased TSA release and recruitment of APCs induced by PTA may also contribute to the proliferation and activation of Th9 cells. Further study is needed to validate this conjecture.

Our study found that PTA combined with adoptive Th9 cell transfer therapy significantly augmented the cytotoxicity of CD8^+^ T cells, surpassing the effects of PTA or Th9 cell monotherapy. However, the functions of CD8^+^ T cells were not enhanced by PTA alone, suggesting that the substantial promotion of their anti-tumor effects induced by combinatorial therapy may, even more, rely on the increased Th9 cells. This hypnosis is further supported by a parallel observation of decreased tumor-infiltrating Th9 cells and compromised CD8^+^ T cell efficacy in the mice model of combinatorial therapy administrated with IL-βRa. Previous studies have elucidated that Th9 cells and their secreted cytokines could (1) directly promote the proliferation and cytokine production of CD8^+^ T cells and (2) consequently induce tumor infiltration and activation of CD8^+^ T cells via recruiting DCs [15, 40, 46]. In addition to IL-9, Th9 cells stimulated by IL-1β also produce IL-21, which significantly promotes the cytotoxicity of CD8^+^ T cells [22]. Nevertheless, further investigation is still required to reveal the detailed mechanism of combinatorial therapy in promoting anti-tumor efficacies of CD8^+^ T cells in our NSCLC models.

Previous studies have found dual impacts of Th9 cells on the development of various malignancies. Th9 cells could facilitate the progression of hepatocellular carcinoma and many hematological tumors, including chronic lymphocytic leukemia, Hodgkin’s lymphoma, B cell lymphoma, anaplastic large-cell lymphoma, NKT cell lymphoma, and thymic lymphoma [46, 48]. On the contrary, Th9 cells demonstrate potent effectiveness against various solid tumors, such as melanoma, colon cancer, and breast cancer [14, 17, 24, 46, 49]. Nevertheless, a debate persists regarding the role of Th9 cells in the development and metastasis of NSCLC. Purwar et al. observed that adoptive Th9 cell transfer effectively impairs the metastasis of lung adenocarcinoma in mice ^38^. Similarly, Shen et al. found that Th9 cells potently inhibit lung adenocarcinoma growth in mice, and an elevated presence of Th9 cells in the tumor tissues correlates with prolonged survival in NSCLC patients [50]. However, Th9 cells may also induce the epithelial-mesenchymal transition, induce migration, prevent apoptosis, and contribute to the proliferation and metastasis of NSCLC cells, resulting in unfavorable prognoses in NSCLC patients [51, 52]. Although neutralizing IL-9 could hinder tumor development in these studies, the systemic transfer of Th9 cells to mice bearing NSCLC tumors was not conducted, thus leaving the impact of Th9 cells on established tumors undisclosed. In our study, Th9 cells, whether administered alone or in combination with PTA, markedly suppressed the growth, recurrence, and lung metastasis of NSCLC in C57BL/6 mice, with their therapeutic efficacies further reaffirmed in the humanized PDX model. discordant conclusions could be attributed to the differing strategies for cell transfer and the quantities of Th9 cells utilized. Additionally, Th9 cells may exert a dual impact on the NSCLC tumor cells and TME. The endogeneity of tumor-infiltrating Th9 cells and their secreted cytokine IL-9 may contribute to the progression of NSCLC tumor cells during their development. Nevertheless, the adoptive transfer of exogenous Th9 cells has the potential to augment the immune response of CD8^+^ T cells, remodel tumor immunosuppressive microenvironments, and ultimately exhibit substantial therapeutic efficacy against NSCLC. Finally, the discrepant conclusions regarding the correlation between lung Th9 cells and patient prognosis in previous research might be due to the various genetic backgrounds, oncogene status, and disease stages of patients [50, 51]. Taken together, although several studies suggest Th9 cells might be tumor-promoting immune cells, the adoptive Th9 cell transfer dose exhibits robust therapeutic efficacies against the established NSCLC tumors, and the additional combination with PTA could promote its positive anti-tumor effect. Such dual efficacies of Th9 cells can also be observed in hematological malignancies, where Th9 cells are widely considered tumor-promoting while CAR-T9 therapy exhibits potent anti-tumor effects [19, 46, 48]. Further investigation is necessary to determine whether adoptive Th9 cell transfer therapy could induce anti-tumor potent effects in NSCLC patients.

Prior research has established an increase in IL-1β levels in NSCLC patients after PTA, suggesting that the combinatorial therapy may also yield a potent anti-tumor efficacy [12]. Given the higher vascularization of pulmonary compared to *s.c.* tissues, it is plausible that Th9 cells transferred into patients could more effectively infiltrate tumors in an in-situ lung cancer model than in a *s.c.* model. This leads to the hypothesis that a combined therapeutic approach could even exhibit superior anti-tumor effectiveness in the in-situ lung cancer model than in the *s.c.* model. Nevertheless, the limitation of pulmonary localization in PTA application to in-situ lung cancer mice model in this study prevented PTA’s application to in-situ lung cancer, therefore we prompted to select a *s.c.* allograft model for easier establishment and tumor growth measurement. Thus, it is critical to validate the combined therapy’s anti-tumor efficacy in autochthonous NSCLC models, such as KRAS-LSL-G12D and EGFR-L858R-T790M mice [53, 54]. Further investigation using autochthonous NSCLC models and long-term observation of PTA application to in-situ lung cancer with or without Th9 cell therapy in distant metastasis is also crucial. Additionally, blood has been widely recognized as a primary conduit for NSCLC metastasis from the lung to distant organs. Our findings indicate that combining PTA with Th9 cell therapy significantly increases Th9 cells and enhances the anti-tumor activity of CD8^+^ T cells in peripheral blood within the lung metastasis model, outperforming either therapy alone. This suggests that the combined treatment may effectively prevent distant metastasis of lung tumors, and further investigation using autochthonous NSCLC models is needed.

In our study, we have validated that the augmented therapeutic effectiveness of PTA combined with Th9 transfer therapy, compared to monotherapy, predominantly depends on the increased levels of IL-1β. Consequently, the utilization of recombinant IL-1 or an IL-1R agonist to mimic IL-1β’s influence on Th9 cells is likely to result in enhanced anti-tumor efficacy in vivo, offering a potential strategy to overcome several inherent limitations of PTA. These limitations include invasiveness, pain, and restrictions in treating tumors located near vital organs such as the heart, aorta, and pulmonary artery due to high intervention risks [55]. Nonetheless, *i.v.* administration of recombinant IL-1 or an IL-1R agonist potentially entails numerous side effects, including pulmonary and bowel inflammation, as well as allergic reactions [56]. Given this, a thorough investigation into the administration route, dosage, frequency, and timing concerning Th9 transfer is necessary to mitigate side effects and enhance efficacy. Additionally, given the pivotal role of IL-1β in PTA-induced promotion of Th9 cells, it is reasonable to speculate that recombinant IL-1 might elicit comparable effects to tumor-derived cytokines post-PTA. Nevertheless, TSAs released after PTA are recognized by DCs, leading to their activation and maturation [12]. Our findings corroborate that PTA also activates DCs. Notably, activated DCs have been shown to foster differentiation and augment the anti-tumor efficacy of Th9 cells [38, 39]. Hence, TSAs may contribute to synergistic PTA-dependent effects, necessitating further validation and investigation of the underlying mechanisms. Additionally, a thorough comparison between recombinant IL-1 and PTA regarding their promotion of Th9 cells is warranted.

Although adoptive Th9 cell transfer therapy has shown excellent anti-tumor effects against progressive malignancies, it is essential to acknowledge its potential dual impact. The further clinical application of adoptive Th9 cell transfer therapy in treating solid tumors may be limited due to the aforementioned oncogenic potential of Th9 cells, particularly in the context of hematological malignancies. Additionally, Th9 cells have been found to induce many autoimmune diseases, encompassing bowel inflammation, inflammatory skin disorders, allergic diseases, encephalomyelitis, and multiple sclerosis [50, 57–59]. Nonetheless, these potential adverse effects of this promising therapeutic approach have not been systematically assessed in preclinical animal models and human studies, necessitating further safety investigations. In recent years, CAR-T and T cell receptor-engineered T (TCR-T) cell therapy have emerged, demonstrating exceptional tumor-targeting abilities and profound anti-tumor efficacies [60, 61]. Of note, CAR-T9 cells have displayed superior and enduring efficacy than CAR-T1 cells against progressive hematologic malignancy [19]. Moreover, tumor-infiltrating lymphocyte (TIL)-based ACT, where TILs are isolated and enriched from patients’ resected tumor tissues, enhanced and expanded in vitro, followed by delivered back as therapeutic agents, has recently been found to associated with improved tumor specificities, enhanced anti-tumor effects, and less off-target toxicities than ACT based on typical T cells against highly progressive tumors including melanoma, glioblastoma, and pancreatic cancer [62, 63]. Given this, developing CAR-T cells, TCR-T cells, or TILs utilizing T9 (Th9 and Tc9) cells holds promise as strategies for obtaining a Th9 cell-based ACT approach with improved tumor recognition and infiltrating, enhanced therapeutic effects, and less off-target effects against solid malignancies.

## Conclusions

**Fig. 9:**
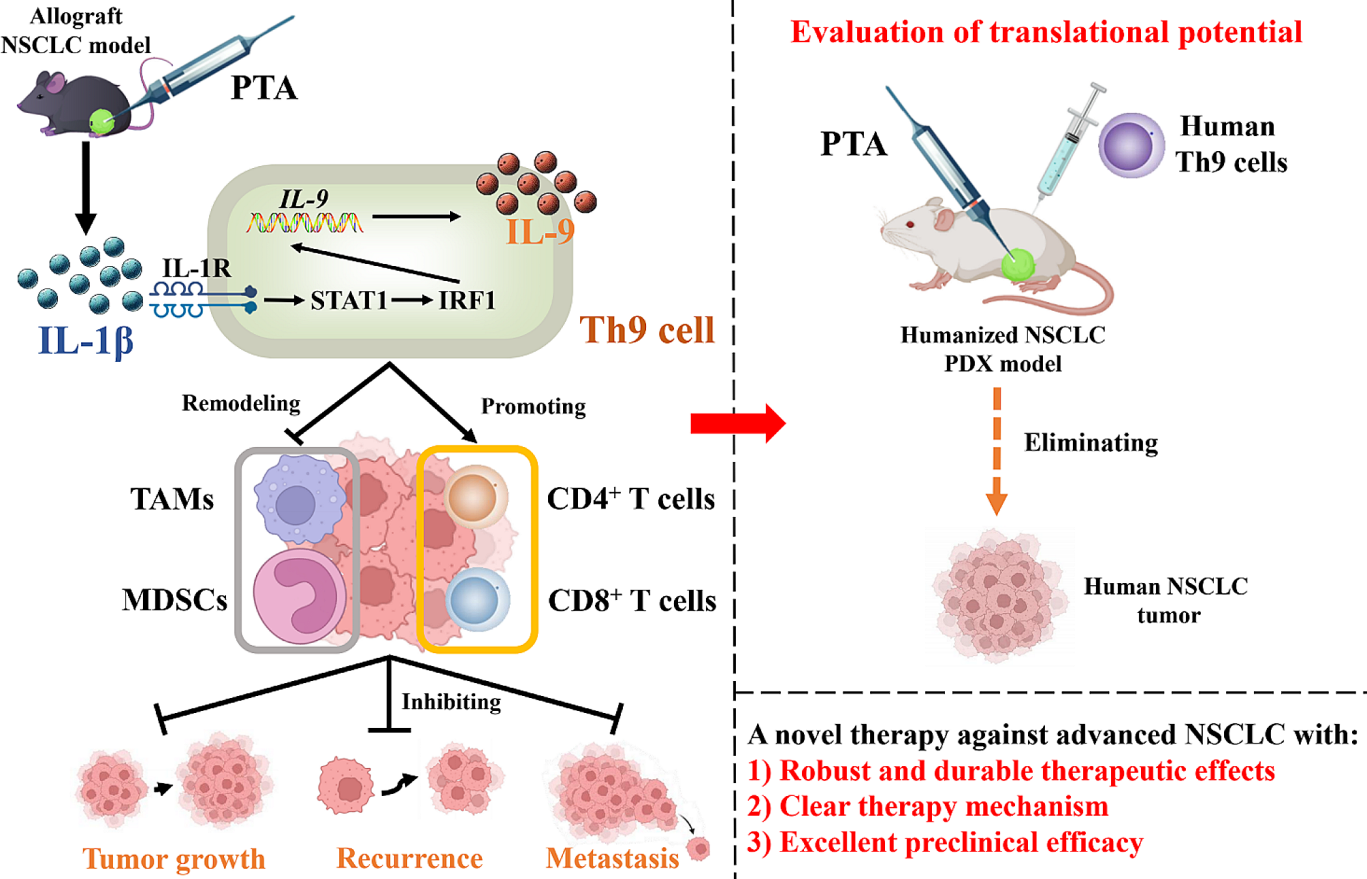
Graphic summary of the study. PTA induces increases IL-1β and further promotes the proliferation and IL-9 production of Th9 cells mainly via the STAT1/IRF1 pathway. The combination of PTA and adoptive Th9 cell transfer therapy exhibits a significantly improved effect on remodeling tumor immunosuppressive microenvironment and enhancing the anti-tumor immune response of T cells and ultimately inhibits NSCLC tumor growth, recurrence, and metastasis and prolongs survival duration of mice. Additionally, combinatorial therapy potently prevents the development of human NSCLC in the humanized PDX mice model and therefore exhibits an excellent translational potential. Taken together, this study develops a novel therapy strategy against advanced NSCLC with robust and durable therapeutic effects, clear therapy mechanisms, and apparent preclinical efficacy. *NSCLC, non-small cell lung cancer; PTA, percutaneous thermal ablation; Th9, T-helper type 9; IL-9, interleukin-9; IL-1β, interleukin-1β; IL-1R, interleukin-1 receptor; TAMs, tumor-associated macrophages; MDSCs, myeloid-derived suppressor cells; PDX, patient-derived xenograft*

In summary, our study illustrated that PTA combined with adoptive Th9 cell transfer therapy exhibits notably enhanced efficacies to enhance anti-tumor effects of CD8^+^ T cells, remodels tumor immunosuppressive microenvironments, and promotes CD8^+^ memory T cells, and ultimately inhibits NSCLC tumor growth, recurrence, and metastasis, leading to extended survival in NSCLC models (Fig. 9). Subsequent mechanistic investigation reveals that PTA stimulates Th9 cell proliferation and IL-9 production by upregulating IL-1β, subsequently activating the STAT1/IRF1 signaling pathway. Finally, we confirm the tumor-eliminating efficacies of combinatorial therapy in humanized NSCLC PDX model. These results indicated that PTA with adoptive Th9 cell therapy holds promise for the treatment of NSCLC, offering robust and enduring anti-tumor efficacy with excellent potential for translation.

### Electronic supplementary material

Below is the link to the electronic supplementary material.

**Additional file 1**: **Table S1**: Primers used for real-time qPCR. **Figure S1**. PTA induces slight and short-lived anti-tumor effects. **Figure S2**. Identifying the IL-9^−^ subgroup of cultured Th9 cells in vitro. **Figure S3**. Combining PTA and adoptive transfer Th9 cell therapy has no synergistic effects on tumor-infiltrating Th1 and Th2 cells. **Figure S4**. Combining PTA and adoptive transfer Th9 cell therapy has no synergistic effects on tumor-infiltrating Tc9 cells. **Figure S5**. Combining PTA and adoptive transfer Th9 cell therapy has no synergistic effects on tumor-infiltrating DCs, B cells, and NK cells. **Figure S6**. PTA promotes Th9 cell differentiation mainly via activating the IL-1β/STAT1/IRF1 pathway and also recruits and activates DCs. **Figure S7**. Combining PTA and adoptive transfer Th9 cell therapy has no synergistic effects on CD8^+^ T_CM_ and T_EMRA_ and CD4^+^ memory T in the TDLNs and spleen, as well as T_RM_ cells in the tumor foci. **Figure S8**. Combining PTA and adoptive transfer Th9 cell therapy has no synergistic effects on Th1 and exhausted CD8^+^ T cells in the TDLNs and peripheral blood.

## Data Availability

All the data in this study are available from the corresponding authors upon reasonable request.

## Abbreviations

NSCLC: Non-small cell lung cancer
ACT: Adoptive cell therapy
PTA: Percutaneous thermal ablation
MWA: Microwave ablation
Th9 cell: T-helper type 9 cells
TSAs: Tumor-specific antigens
TME: Tumor microenvironment
LLC: Lewis lung cancer
IL-9: Interleukin-9
IL-1β: Interleukin-1β
TNF-α: Tumor necrosis factor-α
TGF-β1: Transforming growth factor β1
TAM: Tumor-associated macrophages
MDSCs: Myeloid-derived suppressive cells
Mφ: Macrophages
Tregs: Regulatory T cells
DCs: Dendritic cells
APCs: Antigen-presenting cells
PD-1: Programmed death-1
CTLA-4: Cytotoxic T lymphocyte-associated antigen 4
GITR: Glucocorticoid-induced TNF receptor-related protein
STAT1: Signal transducer and activator of transcription 1
IRF1: Interferon regulatory factor 1
IFN-γ: Interferon-γ
PDX: Patient-derived xenograft
NTG: Nonobese diabetic/ShiLtJGpt-*Prkdc*^em26Cd52^*Il2rg*^em26Cd22^/Gpt
T_EM_ cells: T effector memory cells
TDLNs: Tumor-drainage lymph nodes
*s.c.*: Subcutaneous
*i.v.*: Intravenous
