# Supplementary material for: Combination of percutaneous thermal ablation and adoptive Th9 cell transfer therapy against non-small cell lung cancer
Source: Exp Hematol Oncol. 2024 May 17;13:52. doi: 10.1186/s40164-024-00520-8 (PMC11100251; doi:10.1186/s40164-024-00520-8)
Supplement: Supplementary file 1 — Additional file 1: Table S1: Primers used for real-time qPCR. Figure S1. PTA induces slight and short-lived anti-tumor effects. Figure S2. Identifying the IL-9− subgroup of cultured Th9 cells in vitro. Figure S3. Combining PTA and adoptive transfer Th9 cell therapy has no synergistic effects on tumor-infiltrating Th1 and Th2 cells. Figure S4. Combining PTA and adoptive transfer Th9 cell therapy has no synergistic effects on tumor-infiltrating Tc9 cells. Figure S5. Combining PTA and adoptive transfer Th9 cell therapy has no synergistic effects on tumor-infiltrating DCs, B cells, and NK cells. Figure S6. PTA promotes Th9 cell differentiation mainly via activating the IL-1β/STAT1/IRF1 pathway and also recruits and activates DCs. Figure S7. Combining PTA and adoptive transfer Th9 cell therapy has no synergistic effects on CD8+ TCM and TEMRA and CD4+ memory T in the TDLNs and spleen, as well as TRM cells in the tumor foci. Figure S8. Combining PTA and adoptive transfer Th9 cell therapy has no synergistic effects on Th1 and exhausted CD8+ T cells in the TDLNs and peripheral blood. [file 40164_2024_520_MOESM1_ESM.docx]

**Supplementary information**

**Combination of** **percutaneous thermal ablation and adoptive Th9 cell transfer therapy against non-small cell lung cancer**

**Hanbo Pan^1†^, Yu Tian^1†^, Siyu Pei^1,2†^, Wanlin Yang^1,2^, Yanyang Zhang^1^, Zenan Gu^1^, Hongda Zhu^1^, Ningyuan Zou^1^, Jiaqi Zhang^1^, Long Jiang^1^, Yingjie Hu^1^, Shengping Shen^3^, Kai Wang^4^, Haizhen Jin^4^, Ziming Li^3^, Yanyun Zhang^2^, Yichuan Xiao^2*^, Qingquan Luo^1*^, Hui Wang^1*^, Jia Huang^1*^**

^1^ Department of Thoracic Surgical Oncology, Shanghai Lung Cancer Center, Shanghai Chest Hospital, Shanghai Jiao Tong University School of Medicine, Shanghai, 200030, China

^2^ Chinese Academy of Sciences Key Laboratory of Tissue Microenvironment and Tumor, Shanghai Institute of Nutrition and Health, University of Chinese Academy of Sciences, Chinese Academy of Sciences, Shanghai, 200030, China

^3^ Department of Oncology, Shanghai Lung Cancer Center, Shanghai Chest Hospital, Shanghai Jiao Tong University School of Medicine, Shanghai, 200030, China

^4^ Department of Central Laboratory, Shanghai Chest Hospital, Shanghai Jiao Tong University School of Medicine, Shanghai, 200030, China

**^†^**Hanbo Pan, Yu Tian, and Siyu Pei have contributed equally to this work.

**Supplementary Table 1.** **Primers used for real-time qPCR**

| **Gene** | **Forward sequence (5’-3’)** | **Reverse sequence (5’-3’)** |
| --- | --- | --- |
| Human *ACTB* | ACTCTTCCAGCCTTCCTTCC | CGTACAGGTCTTTGCGGATG |
| Human *IL9* | CTCTGTTTGGGCATTCCCTCT | GGGTATCTTGTTTGCATGGTGG |
| Human *IFR1* | ATGCCCATCACTCGGATGC | CCCTGCTTTGTATCGGCCTG |
| Mouse *Actb* | CGTGAAAAGATGACCCAGATCA | CACAGCCTGGATGGCTACGT |
| Mouse *Il9* | AACAGTCCCTCCCTGTAGCA | AAGGATGATCCACCGTCAAA |
| Mouse *Stat1* | GCTGCCTATGATGTCTCGTTT | TGCTTTTCCGTATGTTGTGCT |
| Mouse *Stat3* | AATATAGCCGATTCCTGCAAGAG | TGGCTTCTCAAGATACCTGCTC |
| Mouse *Stat5* | CAGCCGTGGGATGCTATTGA | GGGACAGCGGTCATACGTG |
| Mouse *Stat6* | CCTGGTCGGTTCAGATGCTTT | GTGCGGCAAGATGCTGTTTC |
| Mouse *Irf1* | AGGCATCCTTGTTGATGTCC | AATTCCAACCAAATCCCAGG |
| Mouse *Irf4* | CAAAGCACAGAGTCACCTGG | TGCAAGCTCTTTGACACACA |
| Mouse *Pu.1* | TTACAGGCGTGCAAAATGGAA | GACGTTGGTATAGCTCTGAATCG |
| Mouse *Traf6* | GATCGGGTTGTGTGTGTCTG | AGACACCCCAGCAGCTAAGA |
| Mouse *Nfκb1* | AGAGGGGATTTCGATTCCGC | CCTGTGGGTAGGATTTCTTGTTC |
| Mouse *Nfκb2* | AGTGTGCGCTGTGTCTGTAG | GTTCTTCTTGGTTACATGCAGGA |
| Mouse *Gata3* | GGGGCCTCTGTCCGTTTAC | TCCAGCTTCATGCTATCTGGC |
| Mouse *Mp3k8* | AGAATGGCCGCTACCAAATCG | CCCCGAGGAACGAAACCAG |
| Mouse *Eomes* | TTCCGGGACAACTACGATTCA | ACGCCGTACCGACCTCC |
| Mouse *Gzma* | CCTGAAGGAGGCTGTGAAAG | GTTACAGTGGGCAGCAGTCA |
| Mouse *Gzmb* | AGGGGGTACAAGGTCACAGA | CAAGAGTGTTGTCCTTGCTCTCT |
| Mouse *Gzmk* | CCGTGGTTTTAGGAGCACAT | TTTTTGGATCCCAGGTGAAG |
| Mouse *Il1r1* | CATTGCCGGGGATGGAAGTC | CACTGGACCTCGGGTAACTC |


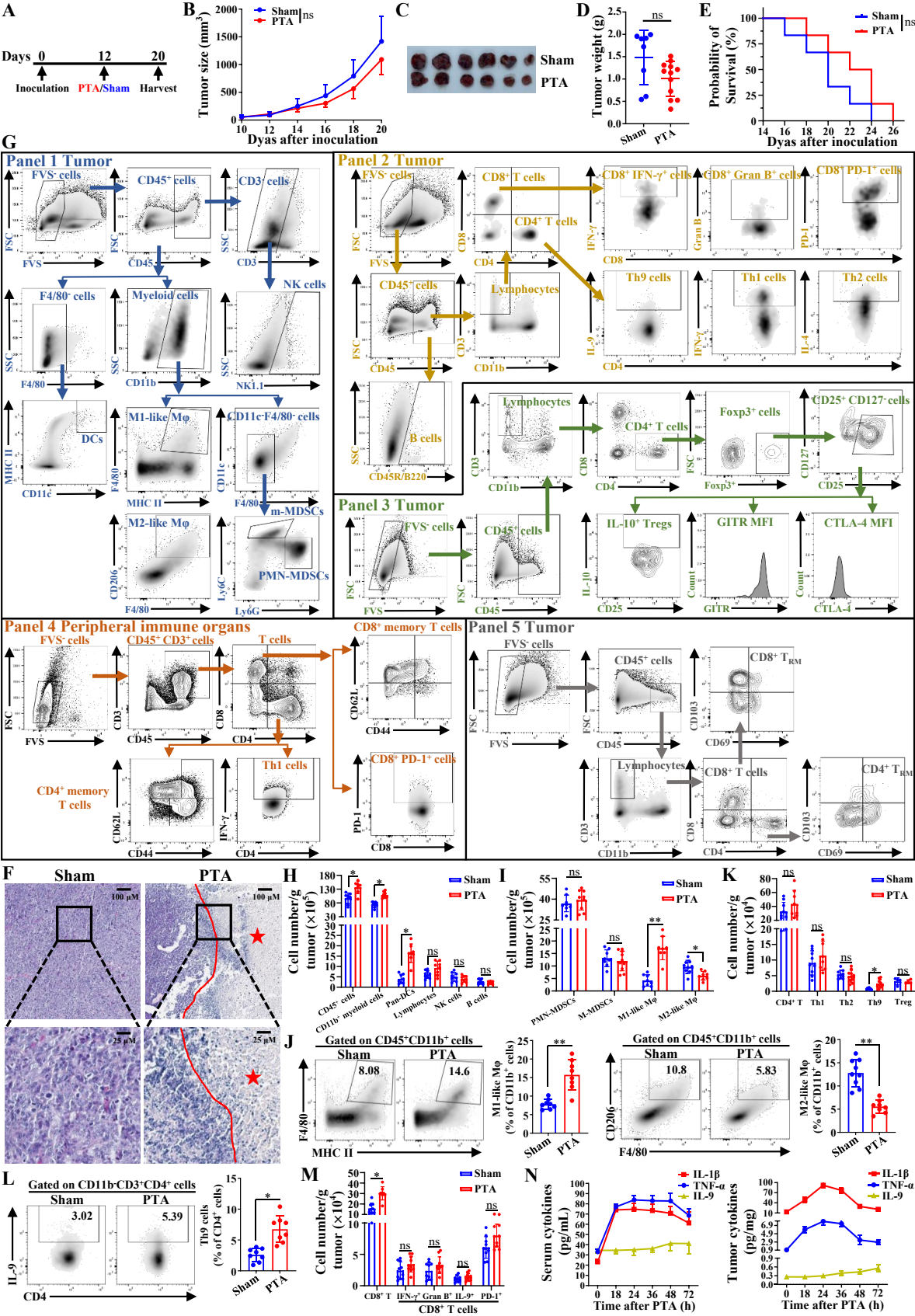


**Supplementary Figure 1. PTA induces slight and short-lived anti-tumor effects.** (**A**) The timeline of treatment. Tumor growth (**B**), representative tumor images (**C**), tumor weights (**D**), Kaplan-Meier survival analysis (**E**)**,** and of C57BL/6 mice that were *s.c.* inoculated with LLC cells and underwent sham or PTA (n = 10-12 mice/group). (**F**) Representative H&E staining images of tumor, as indicated in LLC bearing mice on day 20, as described in (**A**). The “red star” indicated the necrosis areas in the PTA-treated tumor. (**G**) Gating strategy (five main panels) of flow cytometric analysis for tumor-infiltrating and peripheral immune organ immune cells. (**H**-**M**) Flow cytometric analysis of critical tumor-infiltrating immune cells, as indicated in LLC-bearing mice on day 20, as described in (**A**). In (**J**) and (**L**), data are presented as representative plots (left) and summary graphs (right). (**N**) ELISA analysis of the dynamic changes of IL-1β, TNF-α, and IL-9 levels over time (0-72 h) in the serum (left) and tumor (right) of C57BL/6 mice that were *s.c.* inoculated with LLC cells and then underwent PTA. Flow cytometric markers used to define immune cell subtypes (CD45^+^): pan-DCs, F4/80^-^ CD11c^+^ MHC II^+^; lymphocytes, CD11b^-^ CD3^+^; NK cells, CD3^-^ NK1.1^+^; pan-B cells, CD45R/B220^+^; PMN-MDSCs, CD11b^+^ F4/80^-^ CD11c^-^ Ly6G^+^ Ly6C^lo^; m-MDSCs, CD11b^+^ F4/80^-^ CD11c^-^ Ly6G^-^ Ly6C^hi^; M1-like Mφ, CD11b^+^ F4/80^+^ MHC II^+^; M2-like Mφ, CD11b^+^ F4/80^+^ CD206^+^; CD4^+^ T/Th1/Th2/Th9, CD11b^-^ CD3^+^ CD4^+^ IFN-γ^+^/IL-4^+^/IL-9^+^; CD8^+^ T, CD11b^-^ CD3^+^ CD8^+^. Student’s t test was used. Bars, mean; error bars, SD; ^*^, p < 0.05; ^**^, p < 0.01; and ns, not significant.

**
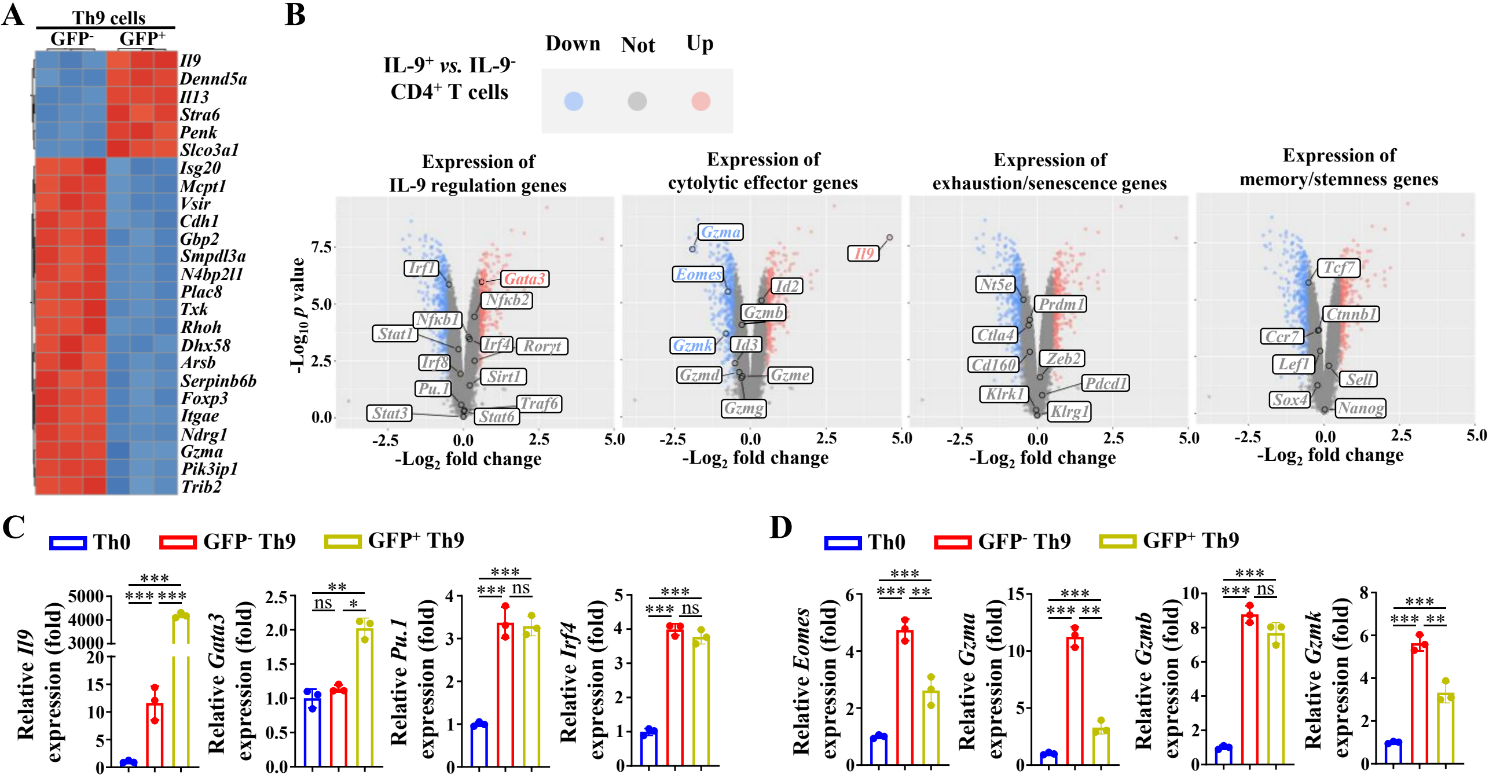
**

**Supplementary Figure 2.** **Identifying the IL-9^-^ subgroup of cultured Th9 cells *in vitro*.** Naïve CD4^+^ T cells from 4-6-week-old IL-9-IRES-EGFP mice were cultured under Th0 or Th9 differentiation conditions for 4 d. GFP^-^ (IL-9^-^) and GFP^+^ (IL-9^+^) cells were isolated by flow cytometry for bulk-RNA sequence (**A**)-(**B**) or qPCR analysis (**C**)-(**D**). (**A**) Heatmap illustrating the top 25 differentiated expressed genes between the GFP^-^ and GFP^+^ cells. (**B**) Volcano plots illustrating the relative expression of gene sets as indicated. qPCR analysis of the mRNA expression of Th9 signature (**C**) and cytotoxic (**D**) genes as indicated. Results were normalized to the expression of *Actb* and are presented in relation to that of Th0 cells. One-way ANOVA with Tukey’s post hoc analysis was used. Bars, mean; error bars, SD; ^*^, p < 0.05; ^**^, p < 0.01; ^***^, p < 0.001; and ns, not significant.


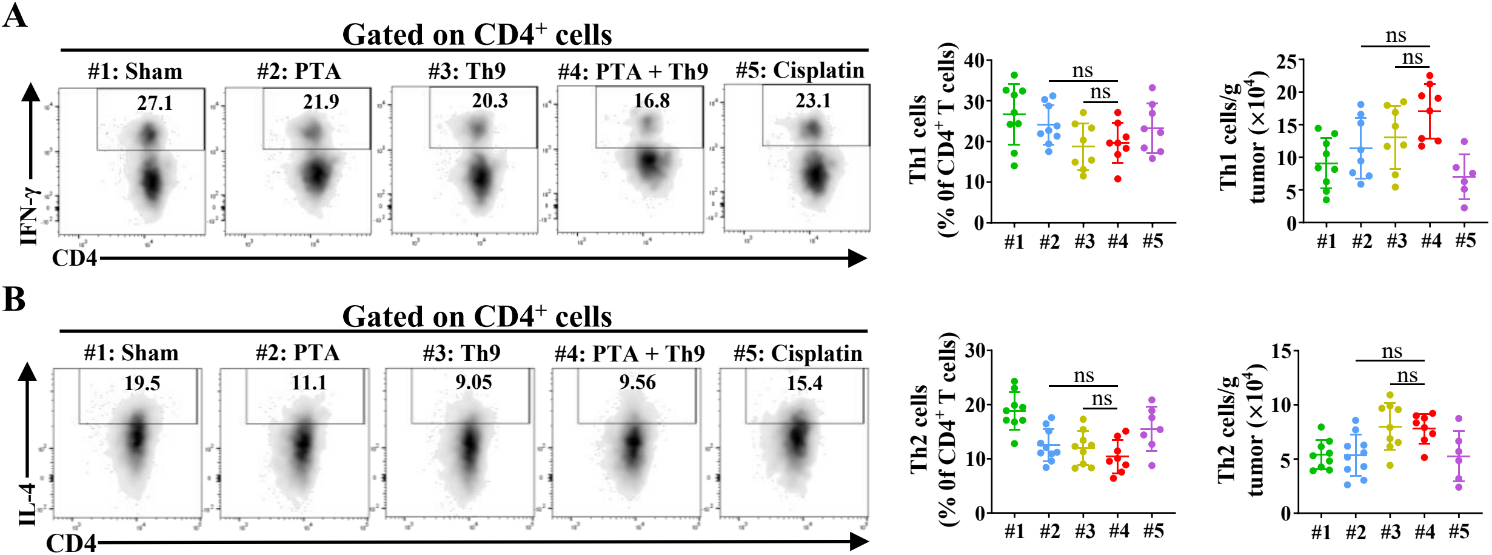


**Supplementary Figure 3. Combining PTA and adoptive transfer Th9 cell therapy has no synergistic effects on tumor-infiltrating Th1 and Th2 cells.** Flow cytometric analysis of tumor-infiltrating Th1 (**A**) and Th2 (**B**) cells, as indicated in LLC-bearing mice on day 20, as described in Figure **1C**. Data are presented as representative plots (left) and summary graphs (right). #1: Sham; #2: PTA; #3: Th9; #4: PTA + Th9; #5: Cisplatin. Flow cytometric markers used to define Th1/ Th2 cells: CD45^+^ CD11b^-^ CD3^+^ CD4^+^ IFN-γ^+^/IL-4^+^. One-way ANOVA with Tukey’s post hoc analysis specified for #2 *vs.* #4 and #3 *vs.* #4 was used. ns, not significant.


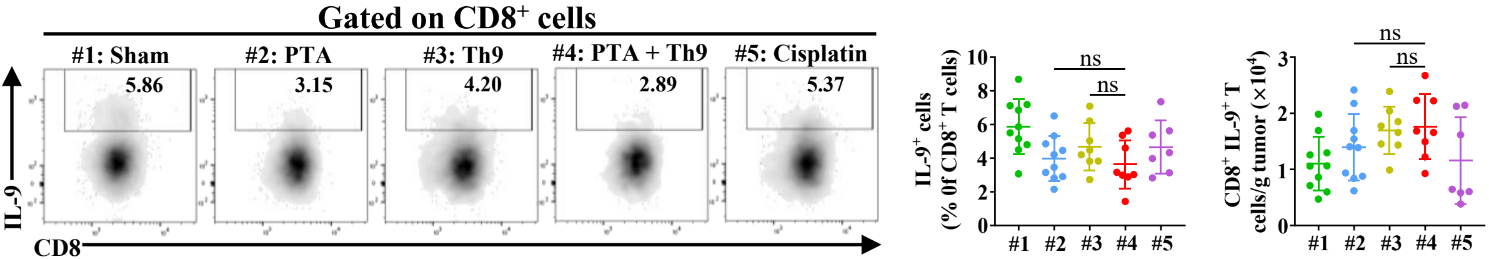


**Supplementary Figure 4. Combining PTA and adoptive transfer Th9 cell therapy has no synergistic effects on tumor-infiltrating Tc9 cells.** Flow cytometric analysis of tumor-infiltrating CD8^+^ IL-9^+^ T cells, as indicated in LLC-bearing mice on day 20, as described in Figure **1C**. Data are presented as representative plots (left) and summary graphs (right). #1: Sham; #2: PTA; #3: Th9; #4: PTA + Th9; #5: Cisplatin. Flow cytometric markers used to define Tc9 cells: CD45^+^ CD11b^-^ CD3^+^ CD8^+^ IL-9^+^. One-way ANOVA with Tukey’s post hoc analysis specified for #2 *vs.* #4 and #3 *vs.* #4 was used. ns, not significant.


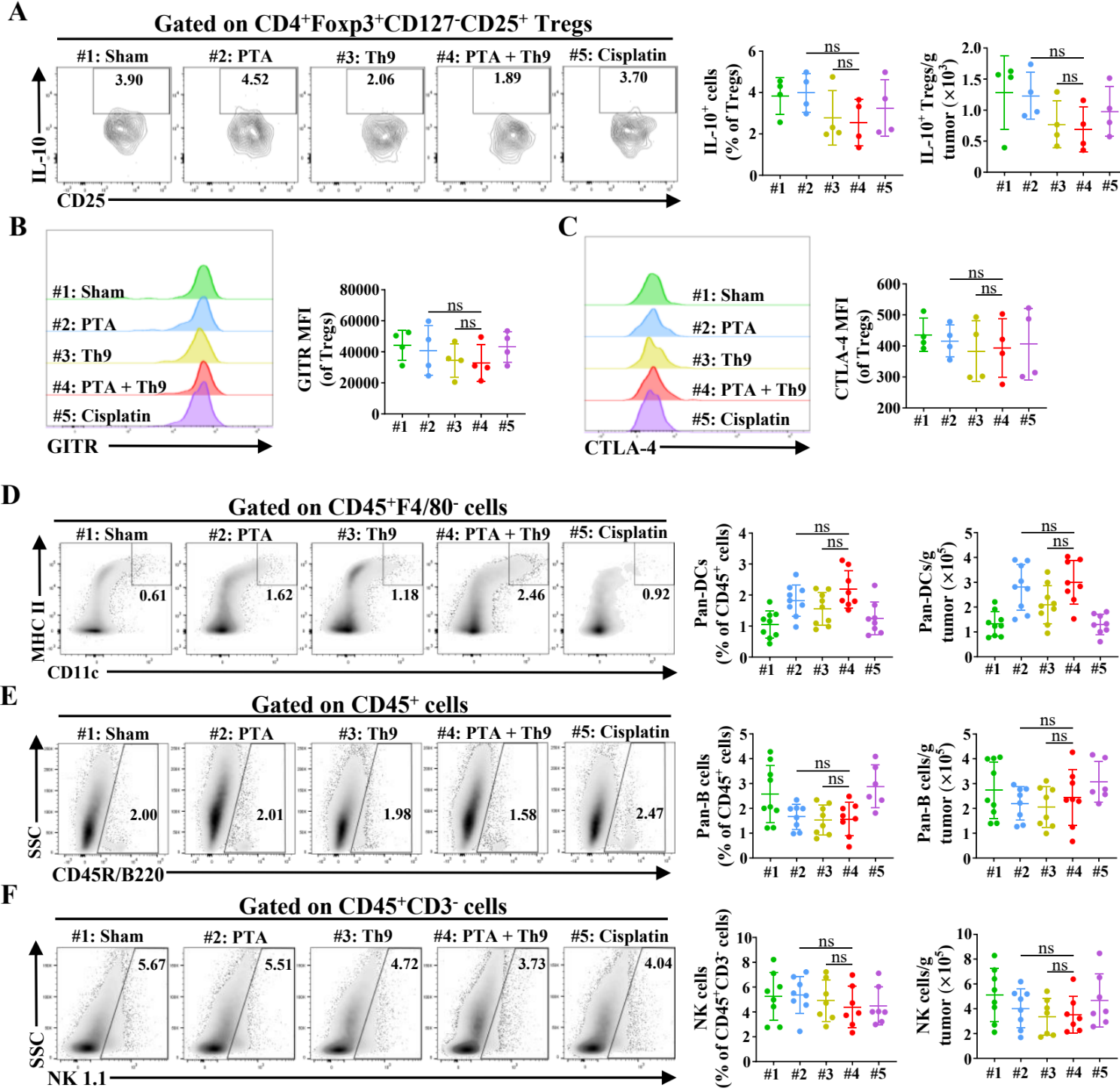


**Supplementary Figure 5. Combining PTA and adoptive transfer Th9 cell therapy has no synergistic effects on tumor-infiltrating DCs, B cells, and NK cells.** Flow cytometric analysis of tumor-infiltrating IL-10^+^ Tregs (**A**), the GITR (**B**) and CTLA-4 (**C**) expression of Tregs, pan-DCs (**D**), pan-B cells (**E**), and NK cells (**F**), as indicated in LLC-bearing mice on day 20, as described in Figure **1C**. Data are presented as representative plots (left) and summary graphs (right). #1: Sham; #2: PTA; #3: Th9; #4: PTA + Th9; #5: Cisplatin. Flow cytometric markers used to define immune cell subtypes (CD45^+^): Tregs, CD11b^-^ CD3^+^ CD4^+^ Foxp3^+^ CD25^+^ CD127^-^; pan-DCs, F4/80^-^ CD11c^+^ MHC II^+^; pan-B cells, CD45R/B220^+^; NK cells, CD3^-^ NK1.1^+^. One-way ANOVA with Tukey’s post hoc analysis specified for #2 *vs.* #4 and #3 *vs.* #4 was used. ns, not significant.

**
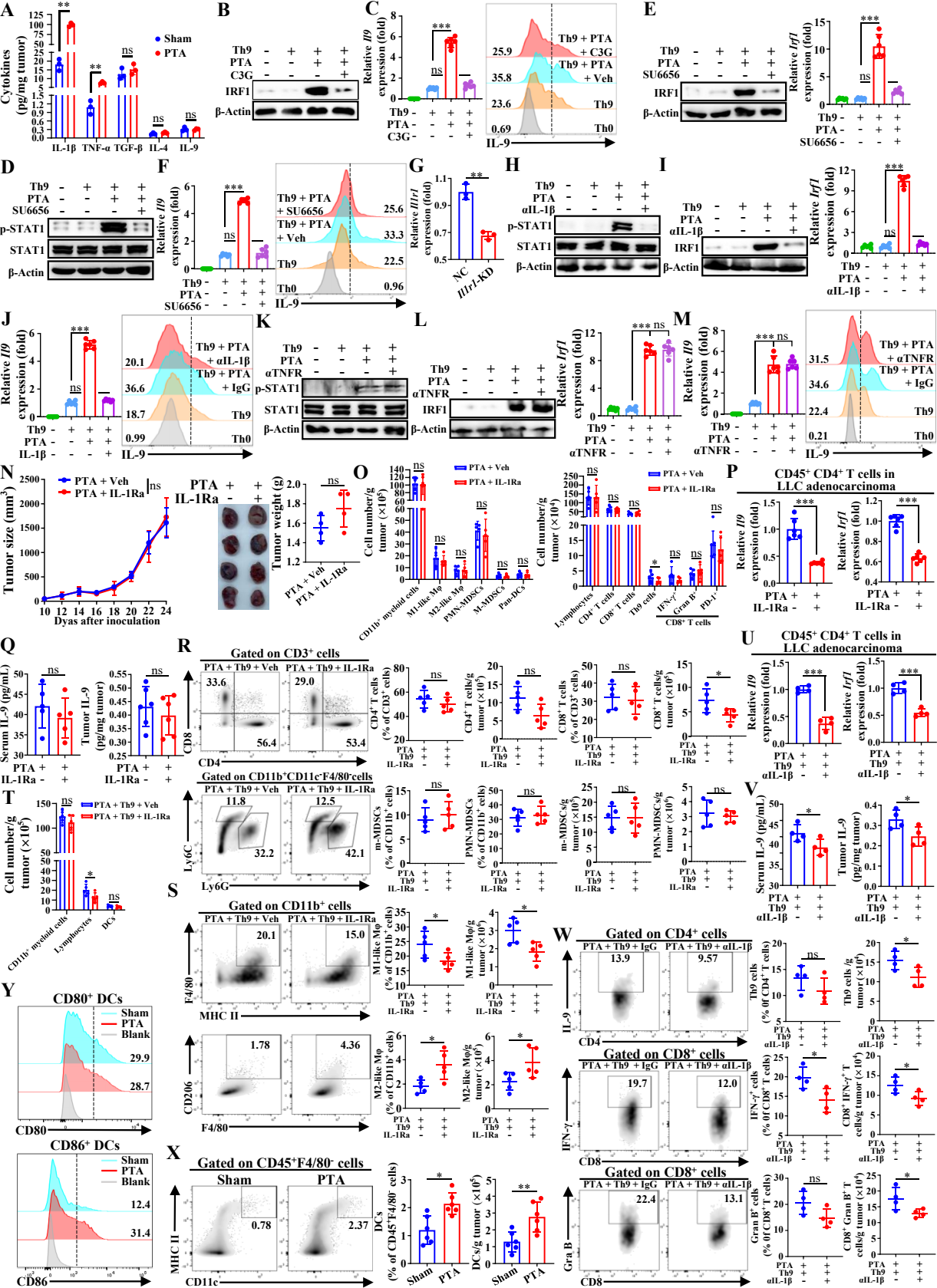
**

**Supplementary Figure 6. PTA promotes Th9 cell differentiation mainly via activating the IL-1β/STAT1/IRF1 pathway and also recruits and activates DCs.** (**A**) ELISA detection of IL-1β, TNF-α, TGF-β1, IL-4, and IL-9 levels of tumor lysate supernatants from 24 h-postoperative LLC-bearing mice underwent sham or PTA. In (**B**-**F**) naïve CD4^+^ T cells were cultured under Th0 or Th9 conditions in the presence or absence of the tumor lysate supernatants from 24 h-postoperative LLC-bearing mice underwent sham or PTA, and qPCR results were normalized to the expression of *Actb* and are presented in relation to that of the control Th9 cells. WB analysis of IRF1 expression (**B**), and qPCR (left) and flow cytometric (right) analysis of IL-9 expression (**C**) in Th9 cells cultured for 72h, with or without C3G. (**D**) WB analysis of p-STAT and STAT expressions in Th9 cells cultured for 25 mins, with or without SU6656. WB (left) and qPCR (right) analysis of IRF1 expression (**E**), and qPCR (left) and flow cytometric (right) analysis of IL-9 expression (**F**) in Th9 cells cultured for 72h, with or without SU6656. (**G**) qPCR analysis of *Il1r1* expression in CD4^+^ T cells transfected with nonsense control shRNA (NC) or *Il1r1*-shRNA. qPCR results were normalized to the expression of *Actb* and are presented in relation to that of the control Th9 cells. (**H**) WB analysis of p-STAT and STAT expressions in Th9 cells cultured for 25 mins, with or without αIL-1β mAb. WB (left) and qPCR (right) analysis of IRF1 expression (**I**), and qPCR (left) and flow cytometric (right) analysis of IL-9 expression (**J**) in Th9 cells cultured for 72h, with or without αIL-1β mAb. (**K**) WB analysis of p-STAT and STAT expressions in Th9 cells cultured for 25 mins, with or without αTNFR1 plus αTNFR2 mAbs. WB (left) and qPCR (right) analysis of IRF1 expression (**L**), and qPCR (left) and flow cytometric (right) analysis of IL-9 expression (**M**) in Th9 cells cultured for 72h, with or without αTNFR1 plus αTNFR2 mAbs. (**N**) Tumor growth, representative tumor image, and tumor weight of C57BL/6 mice were *s.c.* inoculated with LLC cells and then underwent PTA or PTA + IL-1Ra (n = 6 mice/group). (**O**) Flow cytometric analysis of critical tumor-infiltrating immune cells, as indicated in LLC-bearing mice on day 24, as described in (**N**). (**P**) qPCR analysis of *Il9* and *Irf1* expressions in tumor-infiltrating CD45^+^ CD4^+^ T cells that were isolated from LLC-bearing mice on day 24, as described in (**N**). Results were normalized to the expression of *Actb* and are presented in relation to that of the PTA + Veh group. (**Q**) ELISA detection of serum and tumor IL-9 levels in LLC-bearing mice on day 24, as described in (**N**). Flow cytometric analysis of tumor-infiltrating CD4^+^ and CD8^+^ T cells and MDCSs (**R**) and Mφ (**S**), as indicated in LLC-bearing mice on day 24, as described in Figure **5M**. Data are presented as representative plots (left) and summary graphs (right). (**T**) Flow cytometric analysis of tumor-infiltrating myeloid cells, lymphocytes, and DCs, as indicated in LLC-bearing mice on day 24, as described in Figure **5M**. (**U**) qPCR analysis of *Il9* and *Irf1* expressions in tumor-infiltrating CD45^+^ CD4^+^ T cells that were isolated from LLC-bearing mice on day 17, as described in Figure **5S**. Results were normalized to the expression of *Actb* and are presented in relation to that of the PTA + Th9 + IgG group. (**V**) ELISA detection of serum and tumor IL-9 levels in LLC-bearing mice on day 24, as described in Figure **5S**. (**W**) Flow cytometric analysis of tumor-infiltrating lymphocytes, as indicated in LLC-bearing mice on day 17, as described in Figure **5S**. Flow cytometric analysis of tumor-infiltrating total DCs (**X**) and mature (CD80^+^ and CD86^+^) DCs (**Y**), as indicated in LLC-bearing mice on day 20, as described in Figure **S1 A**. Data are presented as representative plots (left) and summary graphs (right). Flow cytometric markers used to define immune cell subtypes (CD45^+^): M1-like Mφ, CD11b^+^ F4/80^+^ MHC II^+^; M2-like Mφ, CD11b^+^ F4/80^+^ CD206^+^; PMN-MDSCs, CD11b^+^ F4/80^-^ CD11c^-^ Ly6G^+^ Ly6C^lo^; m-MDSCs, CD11b^+^ F4/80^-^ CD11c^-^ Ly6G^-^ Ly6C^hi^; pan-DCs, F4/80^-^ CD11c^+^ MHC II^+^; lymphocytes, CD11b^-^ CD3^+^; CD4^+^ T, CD11b^-^ CD3^+^ CD4^+^; CD8^+^ T, CD11b^-^ CD3^+^ CD8^+^; Th9: CD11b^-^ CD3^+^ CD4^+^ IL-9^+^. One-way ANOVA with Tukey’s post hoc analysis or Student’s t test was used. Bars, mean; error bars, SD; ^*^, p < 0.05; ^**^, p < 0.01; ^***^, p < 0.001; and ns, not significant.

**
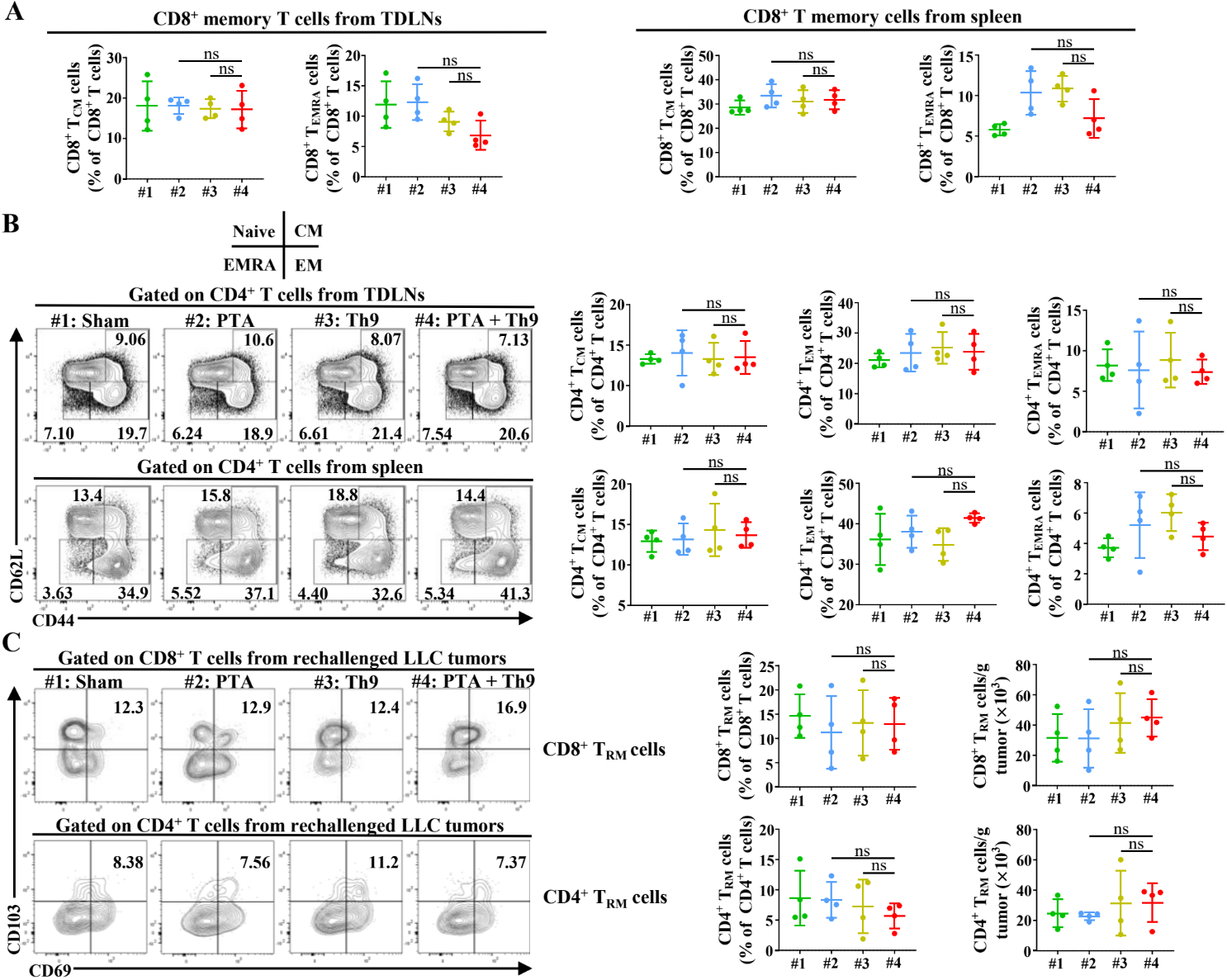
**

**Supplementary Figure 7. Combining PTA and adoptive transfer Th9 cell therapy has no synergistic effects on CD8^+^ T_CM_ and T_EMRA_ and CD4^+^ memory T in the TDLNs and spleen, as well as T_RM_ cells in the tumor foci.** (**A**) The summary graphs of flow cytometric analysis of CD8^+^ T_CM_ and T_EMRA_ cells in the TDLNs (left) and spleen (right), as indicated in LLC/LUC-bearing mice on day 30, as described in Figure **7B**. (**B**) Flow cytometric analysis of CD4^+^ T_EM_, T_CM_, and T_EMRA_ cells in the TDLNs and spleen, as indicated in LLC/LUC-bearing mice on day 30, as described in Figure **7B**. Data are presented as representative plots (left) and summary graphs (right). (**C**) Flow cytometric analysis of tumor-infiltrating CD8^+^ and CD4^+^ T_RM_ cells, as indicated in LLC/LUC-bearing mice on day 30, as described in Figure **7B**. Data are presented as representative plots (left) and summary graphs (right). #1: Sham; #2: PTA; #3: Th9; #4: PTA + Th9. Flow cytometric markers used to define CD4^+^ or CD8^+^ T cell subtypes (CD45^+^ CD11b^-^ CD3^+^ CD4^+^/CD8^+^): naïve T, CD44^lo^ CD62L^hi^; T_CM_, CD44^hi^ CD62L^hi^; T_EMRA_, CD44^lo^ CD62L^lo^; T_EM_, CD44^hi^ CD62L^lo^; T_RM_, CD69^+^ CD103^+^. One-way ANOVA with Tukey’s post hoc analysis specified for #2 *vs.* #4 and #3 *vs.* #4 was used. ns, not significant.

**
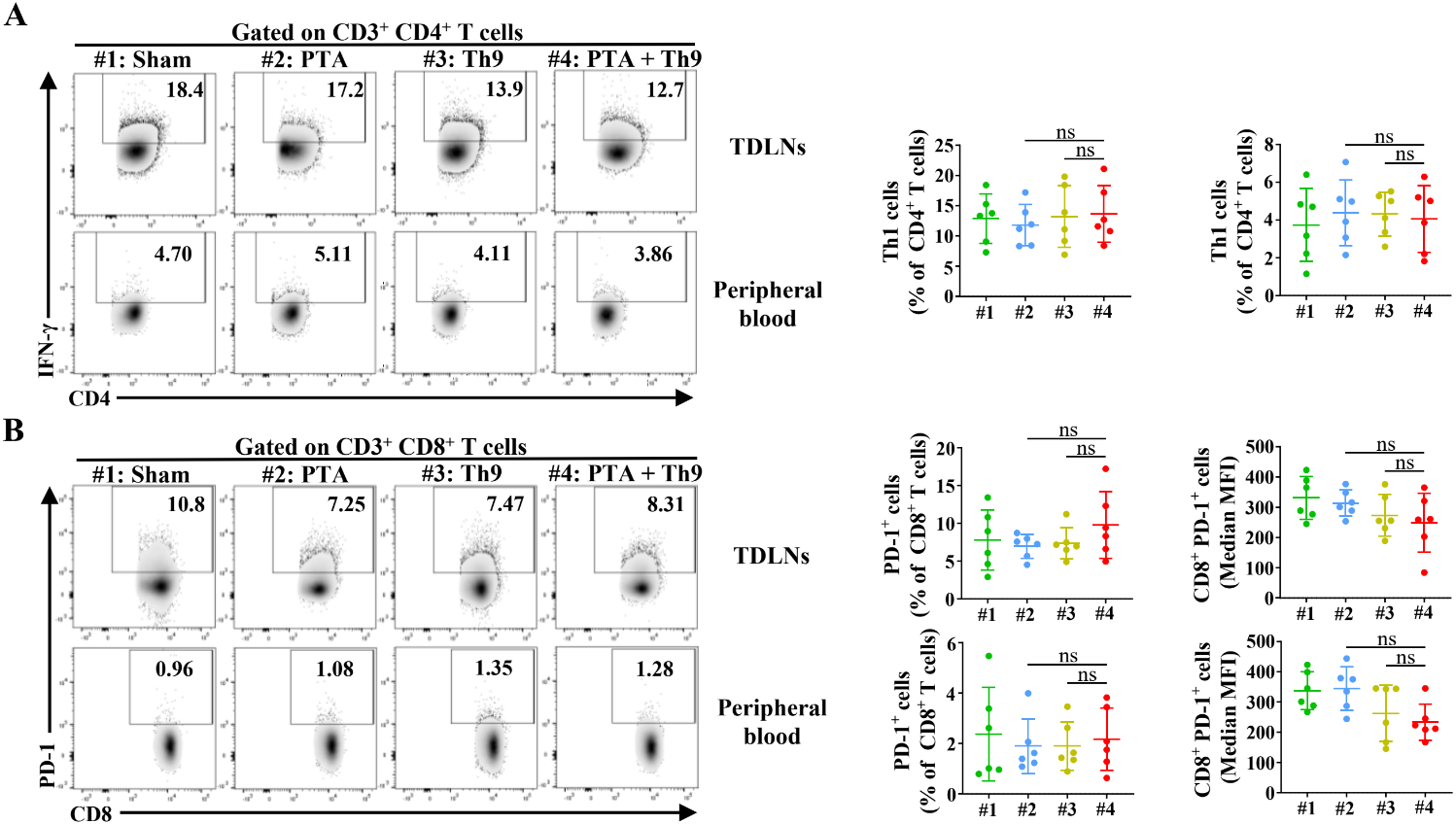
**

**Supplementary Figure 8. Combining PTA and adoptive transfer Th9 cell therapy has no synergistic effects on Th1 and exhausted CD8^+^ T cells in the TDLNs and** **peripheral blood.** Flow cytometric analysis of Th1 cells (**A**), PD-1^+^ CD8^+^ T cells, and PD-1 MFI of CD8^+^ T cells (**B**) in the TDLNs (upper) and peripheral blood (lower), as indicated in LLC/LUC-bearing mice on day 21, as described in Figure **8B**. Data are presented as representative plots (left) and summary graphs (right). #1: Sham; #2: PTA; #3: Th9; #4: PTA + Th9. Flow cytometric markers used to define T cells (CD45^+^ CD11b^-^ CD3^+^): Th1 cells, CD4^+^ IFN-γ^+^; CD8^+^ T cells, CD8^+^. One-way ANOVA with Tukey’s post hoc analysis specified for #2 *vs.* #4 and #3 *vs.* #4 was used. ns, not significant.
